# Review of Applications of Cyclodextrins as Taste-Masking Excipients for Pharmaceutical Purposes

**DOI:** 10.3390/molecules28196964

**Published:** 2023-10-07

**Authors:** Lena Adamkiewicz, Łukasz Szeleszczuk

**Affiliations:** Department of Organic and Physical Chemistry, Faculty of Pharmacy, Medical University of Warsaw, Banacha 1, 02-093 Warsaw, Poland; ladamkiewicz@wum.edu.pl

**Keywords:** cyclodextrins, taste masking, excipients, electronic tongue, inclusion complexes

## Abstract

It is widely recognized that many active pharmaceutical ingredients (APIs) have a disagreeable taste that affects patient acceptability, particularly in children. Consequently, developing dosage forms with a masked taste has attracted a lot of interest. The application of cyclodextrins as pharmaceutical excipients is highly appreciated and well established, including their roles as drug delivery systems, solubilizers and absorption promoters, agents that improve drug stability, or even APIs. The first work describing the application of the taste-masking properties of CDs as pharmaceutical excipients was published in 2001. Since then, numerous studies have shown that these cyclic oligosaccharides can be effectively used for such purposes. Therefore, the aim of this review is to provide insight into studies in this area. To achieve this aim, a systematic evaluation was conducted, which resulted in the selection of 67 works representing both successful and unsuccessful works describing the application of CDs as taste-masking excipients. Particular attention has been given to the methods of evaluation of the taste-masking properties and the factors affecting the outcomes, such as the choice of the proper cyclodextrin or guest–host molar ratio. The conclusions of this review reveal that the application of CDs is not straightforward; nevertheless, this solution can be an effective, safe, and inexpensive method of taste masking for pharmaceutical purposes.

## 1. Opening Remarks

The application of cyclodextrins (CDs) as pharmaceutical excipients is highly appreciated and well established. A lot of review papers focusing on various beneficial properties of CDs have been published, such as their roles as drug delivery systems [1], solubilizers and absorption promoters [2], agents that improve drug stability [3], or even active pharmaceutical ingredients (APIs) [2,4]. However, so far, no reviews have focused on the taste-masking properties of these cyclic oligosaccharides. Therefore, the aim of this work is to provide insight into the studies in this area. Particular attention has been given to the methods of evaluation of the taste-masking properties, and the reviewed cases have been grouped according to the pharmacological groups of the studied APIs. We hope that this article will provide an easy-to-follow overview of the application of CDs as excipients that can be effectively used to mask the unpleasant tastes of APIs.

## 2. Taste Transmission Mechanism in Humans

Prior to addressing flavor-masking strategies, it is important to understand how people transmit taste [5]. Human taste receptors can detect a wide range of substances, but they can only discriminate between five basic tastes: sour, umami, salty, sweet, and bitter [6,7]. There is a distinct channel for each fundamental sense of taste that detects the transfer of flavor signals [6]. G-protein-coupled receptors are triggered by sweetness, umami, and bitterness [8]. Both sour and salty tastes simultaneously communicate taste signals along ionic channels; salty tastes have the potential to activate epithelial sodium channels, but this taste transmission has not received much attention yet [9]. One flavor might influence another taste’s perception if they share the same sort of channel, which may be used in taste masking. For instance, sweets work better to cover up bitterness than acidic substances do [10].

## 3. Taste-Masking Agents as Pharmaceutical Excipients

Many APIs that are commonly used in the pharmaceutical industry have a sour or bitter taste [11]. This is the reason why taking these kinds of drugs can be challenging for some patients, especially children, leading to a decrease in adherence [12]. Therefore, the application of taste-masking agents, defined as substances that improve the drug’s taste and/or smell, is in many cases necessary.

Taste-masking strategies may be broadly divided into two groups based on how tastes are transmitted. The first kind includes the use of flavoring chemicals and bitterness inhibitors to impede taste transmission channels. Flavorings can aid in mitigating the unpleasant taste of medication by competing with the medication to excite taste receptor cells (TRCs) [13]. The second technique involves employing several substances, such as ion-exchange resins, solid dispersions, polymer coatings, prodrugs, microcapsules, liposomes, and nanoemulsions, to prevent the release of the unpleasant-tasting medication into the oral cavity. One of the techniques in this second category is the use of cyclodextrins to disguise tastes. Regardless of the mechanism of action, the requirements for taste-masking agents are strictly defined. They can only be used to mask the bitter taste of substances, and they cannot interact with other compounds or exhibit pharmacological properties [14]. While they are usually solid organics, taste-masking agents can also be used in liquid form: for instance, a pharmacopeial simple syrup is an aqueous solution containing 64% sucrose, commonly applied as an excipient. The high concentration of sugar masks the unpleasant taste of APIs. Additionally, the increased density of the solution keeps the bitter substance dispersed, limiting its contact with taste buds. Furthermore, the simple syrup does not interact with the ingredients of the drug, and it is easy to prepare [15]. Other substances that have similar properties to simple syrup and are commonly used as excipients are mannitol, sorbitol, glycerol, aspartame, and saccharin sodium salt.

Among the substances that improve the taste, there are also those that have flavoring properties that also eliminate the smell of the drug. These mainly include essential oils (rose, dill, anise, lemon, mint) or flavored waters. What is more, concentrated fruit juices (raspberry, cherry, or tinctures from oil raw materials) are also being used for such purposes [16]. 

Other methods of masking the taste or smell of an API include using an insoluble form of the drug, emulsification, the addition of small amounts of anesthetics to block the taste nerve endings, such as sodium phenolate or menthol, and the addition of effervescent substances that anesthetize the taste buds during the release of carbon dioxide.

## 4. Cyclodextrins as Taste-Masking Agents

As stated above, one of the methods that can be used to mask the taste of an API is based on the formation of its complexes. The molecular explanation behind this application is multiform. Firstly, complexes may not exhibit an affinity toward taste receptors, in contrast to the noncomplexed API, due to their apparent structural differences, including the size and shape of the molecule. In addition, the delayed release of the drug into a solution delays the bitterness perception. Further, the slowed release could result in lower quantities of the API present in the mouth that are below the bitterness threshold of the particular API. Cyclodextrins are the perfect choice for such purposes due to their ability to form host–guest inclusion complexes with a wide variety of molecules.

The industrial use of CDs has experienced a sharp rise in interest since the 1970s [17]. This expansion has been accompanied by a clear confirmation of CDs’ nontoxicity and a significant drop in their price. In 1957, when French mistakenly labeled CDs as “toxic” [18], they were claimed to be hazardous. Thankfully, Szejtli proposed the absence of toxicity, a hypothesis that was carefully researched and, in the end, broadly accepted [19].

**Figure 1:**
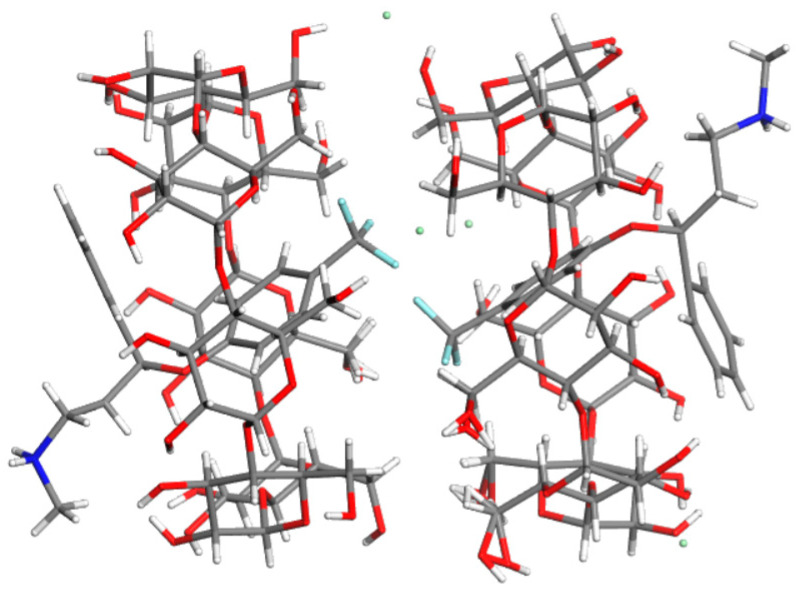
Crystal structure of the inclusion complex formed between β-cyclodextrin and fluoxetine hydrochloride, one of the APIs whose taste was successfully masked using CD. CCDC Refcode BARDEH [21].

Due to their unique features, CDs are largely employed in medicinal compositions [20]. By forming host–guest complexes, they increase the solubility of poorly soluble drugs and protect molecules from environmental influences, including light, humidity, and heat (Figure 1). The beneficial characteristics of CDs in terms of increasing the solubility of guest molecules (i.e., APIs) can be explained at the molecular level. CD molecules have a “donut” ring shape that may trap small, usually non-polar substances. Due to the presence of hydroxyl groups, the exterior parts of CD molecules are polar. The host–guest complex created when an API enters the cyclodextrin molecular hole is polar and is consequently more soluble than the isolated guest molecule.

Large-ring cyclodextrins (LR-CDs), which range in size from nine to more than several hundred units, are also being researched and employed, although the three CDs that are most frequently used are those that contain six, seven, and eight glucose subunits [22]. In addition to native (non-substituted) CDs, the food industry, medicines, cosmetics, biomedicine, and textiles have all found significant usage for these derivatives [23] (Table 1).

## 5. Taste Assessment Methods

The outcome of a flavor evaluation experiment serves as a reference point for evaluating taste-masking technology and modifying manufacturing settings. It is therefore crucial to go into depth about the frequently utilized taste evaluation tools and procedures. The most common methods used to evaluate the taste-masking abilities of cyclodextrins are (1) the employment of volunteers who recognize the flavors of the substances and (2) an electronic tongue.

**Figure 2:**
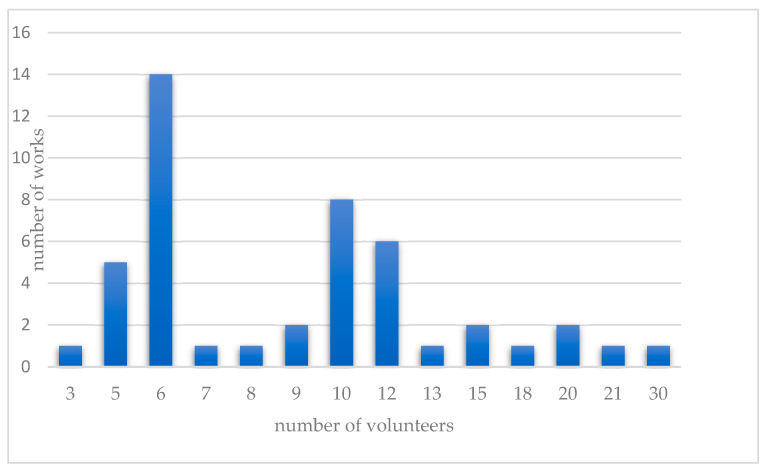
Number of volunteers participating in the reviewed works analyzing the taste-masking properties of cyclodextrins.

The first method includes healthy volunteers, usually both male and female, in the age range of 18–63 (usually n = 3–30, Figure 2), who rate the bitterness of the substances on a scale, e.g., of 0–6.25, using the following scale: 0 ¼ tasteless, 1 ¼ very slightly bitter, 2 ¼ slightly bitter, 3 ¼ moderately bitter, 4 ¼ moderately to strongly bitter, 5 ¼ strongly bitter, 6 ¼ very strongly bitter. Other scales, e.g., 1–5, are also encountered (Table 2) [24].

Most frequently, the volunteers are asked to randomly take one tablet and keep it on their tongues for a few seconds. Tablets may include the analyzed substances or their complexes with cyclodextrins. Before the experiment, the volunteers are usually asked to drink a cup of water. They may be asked to score the formulations’ first taste, aftertaste, mouthfeel, flavor, and general acceptability during the taste test.

**Figure 3:**
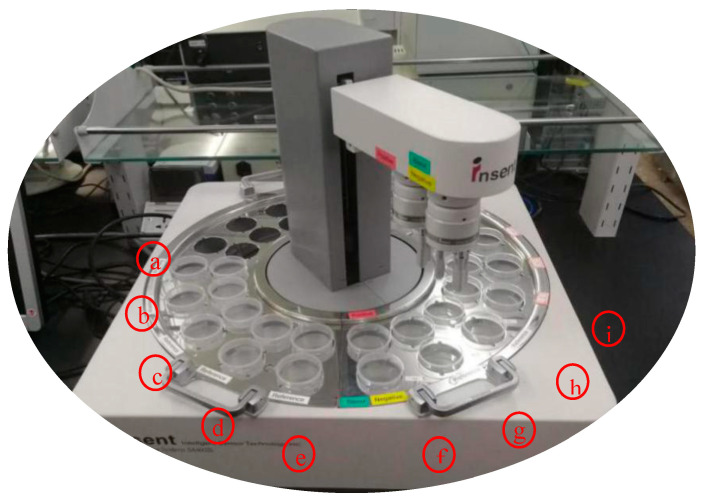
The SA-402B e-tongue system (a is used to measure the aftertaste value, b–c are used to quickly clean the sample, d–e are used to clean the positive and negative solutions, f is the positive and negative cleaning solutions, g is applied for sensor calibration, h is used for sensor reset, and i is the sample). Adapted from [28], licensed under CC BY 3.0.

The second method to evaluate the taste-masking abilities of cyclodextrin involves the use of a device called an electronic tongue (Figure 3). An analytical tool called an “Electronic Tongue” (or “e-tongue”) consists of a number of non-specific, imperfectly selective chemical sensors that are only partially specific to various components in a solution, as well as a suitable system for pattern recognition and/or multivariate calibration for data processing. The stability of sensor behavior and improved cross-sensitivity, which is interpreted as a consistent response of a sensor to as many species as feasible, are of the utmost significance. The e-tongue can recognize the quantitative and qualitative compositions of multicomponent solutions of various natures if correctly designed and calibrated. The e-tongue is a prospective analytical instrument to evaluate the masked bitterness of pure medicinal substances by non-medicinal components [26,27].

The application setup required to perform an analysis using an electronic tongue consists of preparing the solution at a constant temperature (25 °C). The next step is loading the solution (10–100 mL) into the e-tongue test beakers. The seven-sensor assembly and reference electrode are then submerged into each test beaker for an acquisition period (i.e., 120 s). To avoid cross-contamination or the carryover of residues from earlier samples, two rinse beakers, each containing new non-deionized distilled water, are then sequentially filled for 10 s. This series is repeated a couple of times (usually 4–9 times) in rotation. The e-tongue software measures and records the potential difference that is produced between each individual sensor and the reference electrode. 

In the reviewed works, we have found only one in which these two methods of taste evaluation were used at the same time [29]. The authors asserted that simultaneous analysis by these two methods is beneficial for the study; while the human taste trial validates the acceptance of the chosen potential formulations, the electronic taste-detecting system (e-tongue) data may be utilized to give direction on the selection of flavor-masked formulations. Finally, it should be noted that there are many other methods for evaluating the taste-masking effect, such as facial expression and organoid-based methods, in addition to electronic tongue and human volunteer tasting. However, so far, they have not been used in studies including CDs as excipients.

## 6. Overview of APIs Complexed with CDs to Mask Their Taste

The APIs that were chosen to create complexes with cyclodextrins in order to remove their bitter taste were from several pharmacological groups. Most of them were from R06, antihistamines for systemic use (13). The most popular ones were meloxicam (4) from M01, cetirizine dihydrochloride (4) from R06, and levocetirizine dihydrochloride (4) from R06.

Below is a list of APIs used to prepare complexes with CDs, grouped by their ATC (Anatomical Therapeutic Chemical) classification (https://www.whocc.no/atc_ddd_index/ (accessed on 26 July 2023)). The numbers in the brackets indicate the number of studies devoted to the particular API.

- A02: ranitidine hydrochloride (1), famotidine (3);
- A03: propantheline bromide (1), oxyphenonium bromide (1);
- A07: prednisolone (1), loperamide hydrochloride (1);
- A16: 4-phenylbutyrate (1);
- C01: lidocaine hydrochloride (1), Indomethacin (1);
- C03: furosemide (1);
- C05: diltiazem hydrochloride (1);
- C09: captopril (1);
- D04: promethazine hydrochloride (2);
- D08: triclosan (1);
- G04: vardenafil (1);
- J01: cefixime trihydrate (2), lomefloxacin hydrochloride (1), cefuroxime axetil (3);
- J05: oseltamivir phosphate (1);
- M01: meloxicam (4), lornoxicam (1), ibuprofen (1), aclofenac (1);
- N02: rizatriptan benzoate (1), sumatriptan succinate (1);
- N03: lamotrigine (1), gabapentin (1);
- N05: aripiprazole (1), hydroxyzine (1);
- N06: fluoxetine (1), donepezil (1), paroxetine hydrochloride (1), atomoxetine hydrochloride (1);
- P01: primaquine phosphate (1), artemether (1);
- R05: dextromethorphan hydrobromide (1);
- R06: cetirizine hydrochloride (3), cetirizine dihydrochloride (4), levocetirizine dihydrochloride (4), diphenhydramine epinastine (1), DL-chlorpheniramine (1);
- Others: bromelain hydrolysate (1), chitosan (1), allicin (1), arundic acid (1), bitterness suppressants of berberine hydrochloride (1).

Apart from pure APIs, sometimes mixtures or plant extracts are also used for this purpose, for example, goat’s milk, soybean meal, bitter gourd extract (BGE), and beany off-flavors in plant-based meat analogs. The complete list of reviewed works, together with additional information, can be found in Table 3.

## 7. Detailed Review of the Selected Examples

While the list presented in Section 6 and Table 3 reveal the complete index of APIs that have been complexed in order to mask their unpleasant tastes, in this section, the most interesting examples will be described in more detail. To increase the clarity, the complexed APIs have been grouped according to their ATC classifications.

### 7.1. Alimentary Tract and Metabolism Drugs (A)

**Famotidine**: In [32], the authors investigated the potential of a ternary system (comprising famotidine, β-CD or its derivatives, and a hydrophilic polymer) as an approach for enhancing the aqueous solubility and masking the bitter taste of famotidine. The presence of modified β-CDs, notably sulfobutyl ether β-CD (SBE-β-CD), increased the solubility of famotidine in water, and the combination of SBE-β-CD and polyvinyl pyrrolidone (Povidone) K30 increased this solubility even more. By using kneading and freeze-drying techniques, solid binary (drug-SBE-β-CD) and ternary (drug-SBE-β-CD-Povidone K30) systems were created. These solid complexes have substantially faster rates of dissolution than the API by itself. To test the ternary complexation’s capacity for taste-masking capabilities, a taste perception investigation was conducted first by using a taste-sensing device and then by using volunteers. The findings showed that SBE-β-CD and Povidone K30 work well together to increase famotidine’s solubility and rate of dissolution as well as to mask its harsh taste. This beneficial effect was achieved as a combination of two factors. First, the solubility of famotidine in the studied formulation was higher than that of pure famotidine due to the presence of CD. Also, the delay in the release caused by the presence of the polymer prevented the contact of the API with the taste buds, which was the reason behind the successful taste masking.

Also, in [31], the authors evaluated the potential of ternary complexation (comprising the drug, cyclodextrin, and a polymer) as an approach for taste masking. To assess the potential of ternary complexation (a method for masking taste that combines a medication, cyclodextrin, and polymer), famotidine, which has a bitter taste, was chosen as a model API. By creating a ternary complex with the hydrophilic polymer hydroxy propyl methyl cellulose (HPMC) as the third component, the improvement in the taste-masking ability of cyclodextrin toward famotidine was examined. Both the binary (drug–cyclodextrin and drug–polymer) and ternary (drug–cyclodextrin–polymer) systems underwent a phase solubility investigation at 25 °C. The ternary complex was created utilizing the solution method, and it was then further examined using microscopic, PXRD, DSC, and FT-IR techniques. In order to ascertain how ternary complexation affects drug release (D.E 15 min = 90%), in vitro dissolution research was carried out. The improved complexation of famotidine in the ternary system as compared to the binary system was thought to be the cause of efficient taste masking, and this was validated by characterization experiments. The study concluded by showing that ternary complexation may be used as a different strategy for efficient flavor masking.

**Loperamide hydrochloride**: In [36], the authors compared two maltodextrins (MDs, dextrose equivalent: MD1-17 and MD2-13) to well-known water-soluble CDs in terms of their solubilizing and taste-masking abilities. Dextromethorphan hydrobromide and loperamide hydrochloride, two APIs with low solubility, were utilized to compare the effects of CDs and MDs. Dextromethorphan hydrobromide and loperamide hydrochloride became more soluble when 5 mM CD or MD was added to an oversaturated API solution. These APIs are considered to have a bitter flavor. Through the use of electronic taste-sensing devices, or “e-tongues”, the taste-masking effects of CDs and MDs on dextromethorphan hydrobromide, loperamide hydrochloride, and the extremely soluble cetirizine hydrochloride were assessed. Principal component analysis was used to analyze the data from the bitterness sensor and provide a relative API complex rating. The analysis showed greater bitterness masking with MD1 for loperamide hydrochloride and dextromethorphan hydrobromide but better bitterness masking with CD for cetirizine hydrochloride. These findings suggest that MDs have superior solubility effects to CDs for all examined APIs and that MDs may have stronger taste-masking properties than CDs for specific APIs. They came to the conclusion that using MDs offers a promising new strategy for creating medication formulations. Similarly to the other studies, an apparent correlation between the enhanced solubility and taste-masking properties was visible. The possible explanation is that the dissolution of the complex is more favorable than the dissolution of the crystalline API, which increases the solubility of the drug. However, it is also possible that the complex formation delays the initial burst effect and hence masks the bitter taste.

**4-Phenylbutyrate**: In [37], the characteristics of the complexation of 4-phenylobutarate (PB) with native CDs, specifically α-, β-, and γ-CDs, in solution, as well as their ability to mask PB’s disagreeable flavor, were assessed. It was demonstrated that mixing PB with the CDs can make the PB less bitter. Notably, the creation of a soluble inclusion complex with PB by α-CD significantly reduced the bitter taste of PB to 30% of the initial response at a 1:1 molar ratio in vitro. Thus, the study offers a solid foundation for the formulation of PB as a flavor-masked CD complex that can be given orally or nasogastrically to urea cycle disorder (UCD) patients of all ages. Therefore, the shortcomings of current PB formulations are addressed by this taste-masking strategy.

### 7.2. Cardiovascular System Drugs (C)

**Diltiazem hydrochloride**: Diltiazem hydrochloride is a drug with a bitter taste. However, [41] showed that by combining the β-CD and freeze-drying methods, it may be successfully taste-masked. Oro-dispersible tablets (ODTs) were made using the taste-masked complex. Doshion P544 (4%), crospovidone (8%), and sodium starch glycolate (4%), used as a superdisintegrant in tablet formulation, exhibited quicker disintegration and drug release. The developed mixture produced notable improvements in taste and bioavailability. **Spironolactone**: In [40], the authors showed that the addition of HP-β-CD results in an increase in the solubility of spironolactone; however, this did not significantly improve the taste of this API. This is most likely because of the weak host–guest interactions, as was confirmed by NMR analysis and molecular docking calculations. These results imply that the inclusion complex formation between the API and CD is a necessary but not sufficient requirement for taste-masking effectiveness when utilizing CDs to cover up the disagreeable taste of a particular API. Although the investigated HP-CD-containing solution did not significantly improve the unpleasant taste of spironolactone, the authors concluded that the physicochemical and computational findings of this study may support the design and preparation of other cyclodextrin-containing oral pediatric formulations, including solid dosage forms (such as spray-dried powders and oro-dispersible mini-tablets).

### 7.3. Dermatological Drugs (D)

**Triclosan**: The goal of this work [45] was to create triclosan (TC)-containing fast-dissolving films for local distribution to the oral cavity. For the purpose of optimizing the composition of quickly dissolving films, several film-forming agents, film modifiers, and polyhydric alcohols were tested. The authors looked into how Poloxamer 407 and HP-β-CD could increase the solubility of TC. The ingredients hydroxypropyl methylcellulose (HPMC), xanthan gum, and xylitol were combined to create fast-dissolving films. The solubility of TC was significantly improved by the use of Poloxamer 407 and HP-β-CD. Fast-dissolving films made with the TC-HP-β-CD complex and TC-Poloxamer 407 were tested for their in vitro microbiological properties and dissolution profiles. In comparison to films containing the TC-HP-β-CD complex, those containing TC-Poloxamer 407 had improved in vitro dissolution profiles and in vitro antibacterial activity. Human volunteers were used to assess how adding eugenol affected the in vivo performance of films containing TC-Poloxamer 407. Films that contained eugenol enhanced the TC-Poloxamer 407 films’ acceptability in terms of flavor masking and tongue refreshing without affecting the in vivo dissolution time. In this study, the authors used two methods of taste masking: the formation of complexes with CD, namely, HP-β-CD, as well as the addition of eugenol. The authors did not attribute the effect to the particular component of the drug formulation.

### 7.4. Genito-Urinary System and Sex Hormone Drugs (G)

**Vardenafil**: One study [46] found that vardenafil’s (VDR) inclusion complex with ß-CD at a 1:2 molar ratio reduced its bitter taste and increased its solubility in water. The newly developed tablet has a masked taste, an appropriate degree of hardness, and a rapid period of disintegration for the medication’s rapid release. The newly prepared VDR complex grants a higher bioavailability than commercially available tablets, according to the pharmacokinetic analysis results. According to in vivo tests, the oral absorption of VRD from ODTs was likewise clearly higher than that from the currently available tablets. Additionally, the t_max_ for the commercially available pills was lowered from 2 h to 1 h, demonstrating the rate at which VRD started working and eventually enhancing patient efficacy.

### 7.5. Anti-Infectives for Systemic Use Drugs (J)

**Cefixime trihydrate**: In [47], cefixime trihydrate’s (CFX) solubility and dissolution were improved as a result of its complexation with β-CD. The complex chosen for the film’s (ODF) formation was created using the freeze-drying technique since it had the maximum drug concentration and an excellent dissolution profile. The films were homogeneous in weight and thickness, smooth, flexible, elegant, and edible and had a sufficient drug content and a short disintegration time. When compared to films without inclusion complexes, the improved formulation of an inclusion complex (C4) releases the API more quickly, as it also contains tween 80, which accelerates drug release. Because of this, the ODF containing CFX with improved solubility and palatability will be a more sophisticated method of oral drug delivery, helping to increase patient compliance, especially in dysphagic patients.

**Cefuroxime axetil**: In this study [50], cefuroxime axetil (CFA) binary and ternary complexes were made using the kneading process. The researchers came to the conclusion that PVP K-30 can function as a ternary component to enhance the medicinal properties of CFA with β-CD. Ternary complexes outperformed binary complexes in terms of the dissolution profile, releasing >85% of the drug within 30 min. This is because basic PVP K-30 was added, which dramatically increased the Ks and CE phase solubility parameters while also interacting with CFA and β-CD via electrostatic interactions and salt production. An in vitro taste-masking study revealed that the ternary complex was effective in masking the drug’s disagreeable taste. As a result, it can be said that ternary systems of CFA with β-CD and PVP K-30 are a workable way to enhance the medicinal properties of cefuroxime axetil.

### 7.6. Musculo-Skeletal System Drugs (M)

**Aceclofenac**: This study’s [59] goal was to improve the taste-masking and solubilizing abilities of β-CD by making aceclofenac acid-soluble taste-masked granules (ASTMGA) using citric acid and mannitol. Citric acid, mannitol, and β-CD combined in ASTMGA enhanced -cyclodextrin’s ability to mask bitter tastes and increased aceclofenac’s solubility both by facilitating the drug’s entrapment within the molecule and by boosting the inclusion complex’s water solubility. The best formulations of FDT including ASTMGA were quickly developed using a general factorial design in order to have quick disintegration, a palatable flavor, and improved drug dispersion in both simulated salivary fluid and simulated stomach fluid.

**Ibuprofen**: In [58], ibuprofen (IB)-β-CD inclusion complex was prepared by spray drying technique with a 1:1 molar ratio. ODTs were created using the direct compression technique and different superdisintegrant ratios of Ac-Di-Sol^®^ and Kollidon^®^ CL. DSC and FT infrared spectroscopy were used to describe the inclusion complex. ODTs’ physicochemical characteristics and rate of dissolution were assessed. The Dissolution Efficiency (DE_60_) and Dissolved Drug Concentration at 60 Minutes (Q_60_) were also determined. IB-β-CD was successful in hiding the acrid taste of IB. The IB-β-CD (1:1) inclusion complex produced using the spray-drying process improved the water solubility and concealed the disagreeable taste compared to the IB flavor by itself.

### 7.7. Nervous System Drugs (N)

**Rizotriptan benzoate**: In [61], in order to hide the bitter taste of rizatriptan benzoate, HP-β-CD was used in the preparation of tablets. By mixing HP-β-CD with rizatriptan, a taste-masked complex was created, which was then tested for drug content and bitterness. Crospovidone was used as a superdisintegrant in the direct compression method used to create the tablets. The weight variation, mechanical strength, wetting duration, water absorption ratio, and in vitro release characteristics of the tablets were all assessed. When compared to the common form of API, the complex demonstrated good taste masking. Data on hardness and friability showed that the tablets had good mechanical strength. The tablets quickly dispersed in 35 to 71 s, with rizatriptan benzoate releasing at a quicker rate. The three-month stability test revealed no appreciable changes in the tablet quality. As a result, the current investigations demonstrated HP-β-CD’s potential for taste masking (Table 4) and for enhancing rizatriptan benzoate’s dissolution profile following complexation.

**Paroxetine hydrochloride**: With the aid of HP-β-CD and the freeze-drying procedure, the bitter API paroxetine hydrochloride may be successfully taste-masked and used to create oro-dispersible films, as was described in [69]. Faster disintegration and drug release were seen in films made with HPMC E-5 (59%), PG (36%), and PVP K-30 (10%) as superdisintegrants. The developed mixture produced notable improvements in taste and bioavailability. Finally, it can be said that taste-masked PXT oro-dispersible films can be successfully created, with PVP K-30 acting as a superdisintegrant and HP-β-CD serving as a taste-masking agent. This makes the films suitable for anxious and depressed patients, as well as bedridden and dysphasic patients.

**Gabapentin**: Due to its short biological half-life of 5–7 h and limited bioavailability (60%), gabapentin was chosen to produce an oral controlled-release dry solution in [64]. Since gabapentin has a harsh taste, an effort was made to cover it up. The goal was to create and assess a controlled-release dry suspension for reconstitution in order to improve bioavailability and reduce the medication’s acridity. Nanosponges made of β-CD were created using the previously described melt process. The nanosponge–drug complexes were analyzed for taste and saturation solubility as well as by FTIR, DSC, and PXRD physicochemical methods. Using a suspension-layering approach, the complexes were coated on Espheres before being covered in ethyl cellulose and Eudragit RS-100. Drug nanocavities were partially trapped by the complexes. In comparison to the pure API, the complexes had significantly decreased solubility (by 25%) in several media. For 12 h, the nanosponge complex’s microspheres displayed the desired regulated release profile. When the reconstituted suspension was stored for 7 days at 45 °C/75% RH, minimal API leakage was seen. The regulated release of the API and improved taste masking were both supplied by the coated polymers and nanosponges, which successfully masked the taste of gabapentin. According to in vivo research, the controlled-release solution has a 24.09% higher bioavailability than the pure medication. As an appropriate controlled-release drug delivery system for gabapentin, the dry powder suspension filled with microspheres of nanosponges complexes can be suggested.

**Donepezil hydrochloride**: The purpose of this study [68] was to create an ODF for donepezil (DP) that is tasty to aid in swallowing and to look into how cyclodextrin affects taste masking based on dynamic processes and in vivo drug absorption. To cover up the bitter taste, DP was complexed with HP-β-CD, and the complexes were then integrated into the ODF using the solvent-casting process. An e-tongue was used to assess the effectiveness of taste masking, and an in vivo investigation was used to examine the pharmacokinetic behavior of DP/HP-β-CD ODF. The optimized film was bioequivalent to DH and, according to the results, was more palatable than the DH film. Phase solubility analysis, FT-IR, DSC, PXRD, and molecular modeling methods were used to determine the molecular properties of the complex. The creation of DP/HP--CD, which was caused by a modest interaction between DP and HP--CD, was attributed to taste masking. The stomach’s acidic environment, which made it easier for DP to be absorbed, reduced the stability of DP/HP-CD. The findings, according to the authors, improved the knowledge of the use of cyclodextrin complexation and offered recommendations for the design of ODFs, particularly for medications with unpleasant tastes. 

### 7.8. Antiparasitic Products, Insecticides, and Repellents (P)

**Artemether**: In order to disguise the bitterness, improve the drug release, and create a stable, palatable formulation of artemether (ARM) specifically for pediatrics, the authors of [72] looked into the inclusion complexation of ARM with β-CD. To investigate the inclusion complexation, a physical combination and kneaded system were created. The gustatory sensation test was used to measure the bitterness score. Additionally, at pH values of 1.2 and 6.8, the physical mixture and kneaded system showed improved drug release. Based on the bitterness score, the 1:20 ratio physical mixture was chosen in order to provide a tasty reconstitutable dry suspension of ARM. The physical mixture prepared as a reconstitutable dry suspension demonstrated perfect bitter taste masking, high flowability, and simple redispersibility. The reconstitutable dry suspension made with pure ARM was evaluated for taste by human volunteers and scored as tasteless with a score of 0. This proved that ARM could be reconstituted as a stable and tasty dry suspension for flexible pediatric dosing utilizing CD inclusion complexation.

### 7.9. Respiratory System (R)

**Figure 4:**
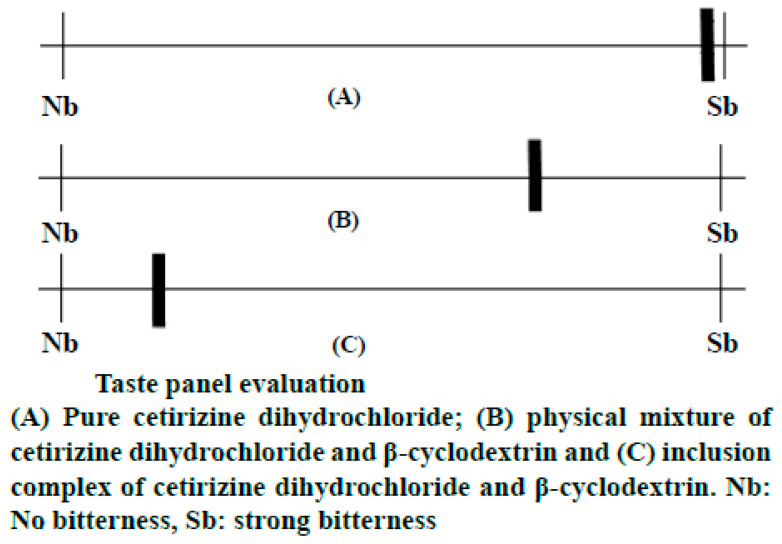
Results of application of β-cyclodextrin to mask the taste of cetirizine dihydrochloride. Even in the form of physical mixture with API, β-CD exhibits some taste-masking properties that are greatly improved when the complex is being administrated. Adapted from [75], licensed under CC BY 3.0 (Creative Commons Attribution License 3.0).

**Cetirizine dihydrochloride**: Cetirizine dihydrochloride complexation with α-, β-, and γ-CDs was studied using nuclear magnetic resonance (NMR), ultraviolet (UV), and isothermal titration calorimetry (ITC) methods in [77]. Overall, the taste of bitterness for the cetirizine-CD solutions was consistent across all examiners. Cetirizine-β-CD solutions had the greatest taste-masking effect, followed by cetirizine-α-CD solutions. When compared to pure cetirizine, the taste-masking impact of cetirizine-γ-CD formulations was the worst. This is most likely caused by the association constant’s low value. The ratio of the CD that was utilized also had an impact on the taste masking; solutions with a molar ratio of 1:5 cetirizine-CD displayed better taste than those with a ratio of 1:2. This is most likely caused by the extra CD, which makes cetirizine primarily complexed. The cetirizine-β-CD solutions, which had a molar ratio of 1:5 cetirizine-CD, had the best taste-masking effects. Of the 13 tasters, 5 and 7 said there was no bitterness or only a mild bitterness, respectively. β-CD’s extraordinary taste-masking ability may be attributed to its sweet flavor and strong connection with the cetirizine molecule, as indicated by its higher association constant than the other two native CDs, α- and γ-CD. The cetirizine-γ-CD solution, which had a 1:5 molar ratio of cetirizine-CD and the weakest taste-masking characteristics, was described as being extremely bitter by 11 out of 13 panelists. Despite the fact that all three native CDs exhibit acceptable complexation with cetirizine, only β-CD is suitable for the formulation of oral pharmaceutical dosage forms (Figure 4).

### 7.10. Others

**Chitosan**: In [82], several gram-scale macromolecular adducts were made by joining chitosan and β- and γ-CDs together through succinyl or maleyl bridges. Their ability to conceal bitter natural extracts (artichoke leaves, aloe, and gentian) was evaluated in a panel test using serial caffeine doses as a reference scale. The highest efficacy was demonstrated by the β-CD-chitosan adduct, and the bitterness mitigation was statistically significant.

**Allicin**: By creating an inclusion with α-CD utilizing a straightforward and environmentally safe manufacturing method, allicin’s disagreeable taste and odor were successfully hidden in [83]. This method of preparation was much easier than the conventional techniques for the preparation of CD inclusion complexes, and it only took 10 min to obtain the desired product. Allicin-α-CD’s stability, which was 33 times better than that of their physical mixture, also experienced significant improvements. The results of molecular dynamics simulations showed that the allicin thiocarbonyl group was partially contained by α-CD, indicating a potential molecular distribution and interaction of allicin-α-CD. It has made a significant improvement in customer compliance and given allicin’s application a fresh, practical approach.

**Arundic acid**: Arundic acid, an oily medication, has a low solubility in water and a strong bitter/irritating taste. To create arundic acid medicinal formulations, these physicochemical qualities must be improved. In [84], the authors showed how arundic acid interacts with native cyclodextrins (CDs) and hydroxypropyl beta cyclodextrin (HP-β-CD), as well as their ability to powderize, dissolve, and conceal tastes. The most effective CD for arundic acid solubilization was HP-β-CD. Studies using UV and ^1^H NMR spectroscopy proved that arundic acid and CDs in solution produced inclusion complexes at a molar ratio of 1:1. The oily form of arundic acid was converted to a solid form through complexation with CDs. According to research on gustatory perception, HP-β-CyD and γ-CD, among other CDs, had the strongest taste-masking effects in solutions and powders, respectively. The response of the electric potential brought on by the adsorption of arundic acid to the taste sensor was greatly diminished by HP-β-CD. These findings imply that multifunctional excipients for creating liquids and powders containing arundic acid may be made from hydrophilic CDs.

**Bitter gourd extract (BGE)**: Although bitter gourd extract (BGE) has several antioxidants and anti-diabetic ingredients that support human health, its bitter flavor makes it difficult to incorporate into cuisine. The authors of [86] examined how carboxymethyl cellulose and β-CD affected the bitterness and other characteristics of BGE. A trained sensory panel assessed the level of bitterness, and the physicochemical characteristics, such as viscosity, total saponin, polyphenol content, antioxidant capacity, and -amylase inhibitory activity, were also identified. The bitterness of BGE with 0.75% w/v β-CD was discovered to have been lowered by more than 90%. Additionally, the creation of a complex between β-CD and components of BGE was confirmed by FTIR, ^1^H NMR, and thermogravimetric analysis. The findings of this study also revealed that taste-masking agents did not inhibit the biological activity of BGE.

## 8. Is Application of CDs as Taste-Masking Agents Successful?

Among the reviewed papers, we have mostly found those in which the application of cyclodextrins as taste-masking excipients was a great success, but also some in which this method was not advantageous. From the successful ones, we can mention the work by Marzouk et al. [67], who prepared complexes of fluoxetine (FLX) with β-cyclodextrin. A group of six volunteers scored the taste of noncomplexed FLX after 10 s as 3 (bitter) and 4 (very bitter). Additionally, they evaluated the taste of the FLX-β-CD complex as 1 (tasteless), indicating a great taste improvement. 

Another successful example of such an application is described in the work by Li et al. [70], who created a complex of atomoxetine hydrochloride with HP-β-CD. While testing the electronic response of HP- β-CD, the authors noticed that only the sensor outputs of AN0 and C00 significantly increased in comparison to those of atomoxetine HCl, which were unchanged. Furthermore, the sensor response was rather slow. As previously stated, each of the three bitterness sensors (AN0, BT0, C00) can successfully identify the bitterness of ionic liquid substances. These results may be explained by the fact that the SA402B taste-detecting system’s chosen sensors are ineffective for neutral substances like HP-β-CD. The taste-sensing system, on the other hand, might be utilized to assess the flavor-masking effectiveness of the formulation with HP-β-CD, given the absence of a reaction to HP-β-CD. Another example of the successful taste-masking properties of cyclodextrins is mentioned in the work by Musuc et al. [42], who prepared a complex of captopril (CAP) and β-cyclodextrin in a 1:2 molar ratio. A taste evaluation was performed by six healthy volunteers who were asked to rank the following taste characteristics from 1 to 5: 0—tasteless; 1—pleasant; 2—slightly sweet; 3—slightly bitter; 4—moderately bitter; and 5—intensely bitter. Three of them judged CAP-β-CD tablets for oral dispersion as grade 1 (pleasant), two of them judged them as grade 2 (slightly sweet), and one judged them as grade 3 (slightly bitter). All of them assessed the oro-dispersible tablets as having a slightly pleasant taste, and no roughness was reported. On the other hand, the CAP tablets had a burning, metallic flavor.

The fourth example of taste-masking success is found in the work by Liu et al. [68], who created a complex between donepezil hydrochloride (DH) and HP-β-CD. An e-tongue assessment was conducted to evaluate the taste of samples using the taste-sensing system SA402B. It was discovered that samples with similar tastes clustered together. The DH-containing samples, physical mixes, DH-ODF, and the DH- and sucralose-containing film accumulated into a cluster, indicating that the flavor was unappealing or that the taste-masking action was ineffective. It was clear from the clustering of donepezil/HP-β-CD inclusion complexes and donepezil/HP-β-CD ODF that cyclodextrin complexation was significantly effective for taste-masking and that the DP/HP-β-CD inclusion complexes were stable in the film.

Also, the work by Al-Gethmy et al. [46], who created a complex of vardenafil with β-CD, should be mentioned. The disintegration time and taste-masking tests were conducted in the buccal cavity of six healthy human participants in a single-blind study. On a scale of 0 to 3, the human test volunteers were asked to judge how bitter the improved recipe tasted. When the score was less than 1, the taste was tolerable, but when it was greater than 1, the tablet’s flavor was bitter and intolerable. The ratings of the six volunteers were not equal or lower until after the complexation, which shows that the formulation has a respectable taste-masking effect. However, there were also some cases in which the formulation with cyclodextrins did not work as designed; for example, we can mention the work by Lopalco et al. [40], who created a complex of spironolactone (SPL) and HP-β-CD. They found that although the complex was formed, the taste did not improve, which was confirmed by a group of 24 healthy volunteers. The results obtained are in line with those from brief-access taste aversion (BATA) experiments. In fact, the rats’ preference for SPL solutions with low HP-β-CD concentrations (1%, 2%, and 6%) grew, while the number of licks decreased for the SPL solution that contained 18% HP-β-CD. Overall, taking into account the BATA experiment’s results and human panel results, HP-β-CD’s taste-masking effect on SPL is not very strong.

Another example of a partly failed taste evaluation is described in the work by Stojanov, Wimmer, and Larsen [77], who prepared complexes of cetirizine dihydrochloride with α-, β-, and γ-CDs. The authors proved the good taste-masking properties of α- and β-CDs, but they noticed that γ-CD did not give efficient results. The reason for this is probably due to the relatively low association constant between the API and this CD. The amount of CD employed also had an impact on flavor masking; for instance, solutions with a cetirizine-CD ratio of 1:5 had better taste than those with a 1:2 ratio. This is most likely caused by the extra CD, which makes cetirizine mostly complexed. The best taste-masking effects were provided by the cetirizine-β-CD solutions, which had a molar ratio of 1:5. Of the 13 tasters, 5 and 7 said there was no bitterness or only a mild bitterness, respectively. β-CD’s extraordinary taste-masking ability may be attributed to its sweet flavor and strong connection with the cetirizine molecule, as indicated by its higher association constant than the other two native CDs (α- and γ-CD). The cetirizine-γ-CD solution, which had a 1:5 molar ratio of cetirizine-CD and the weakest taste-masking characteristics, was described as being extremely bitter by 11 out of 13 panelists. Despite the fact that all three native CDs exhibit acceptable complexation with cetirizine, only β-CD is suitable for the formulation of the oral pharmaceutical dosage form.

Also, the work by Funasaki et al. [34] presents the varied taste-masking capabilities among studied CDs. The authors investigated the mechanisms of masking the bitter taste of propantheline bromide (PB) and oxyphenonium bromide (OB) by native and modified cyclodextrins: α-, β-, and γ-CDs, 2,6-O-dimethyl-β-CD, HP-β-CD, 6-glucosyl-β-CD, and 6-maltosyl-β-CD. Five volunteers were involved in the sensory test. Native and modified β-CD decreased the bitter taste remarkably better than α- and γ-CDs.

**Figure 5:**
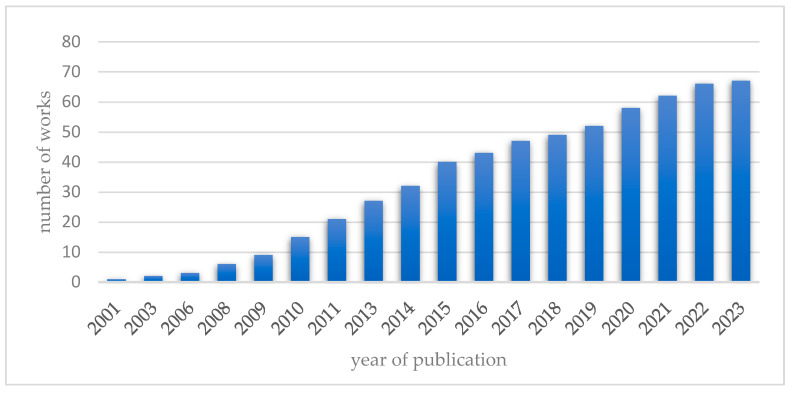
Number of original works describing the taste-masking properties of cyclodextrins. Each column shows the number of articles in the given year and all years before. For example, the column entitled “2010” depicts the number of articles published in the period 2001–2010, including 2010.

Another example of the diverse taste-masking abilities of cyclodextrin is described in the work by Commey et al. [37]. They analyzed the taste-masking abilities of inclusion complexes obtained in solution between 4-phenylbutyrate (PB) and α-, β-, and γ-CDs. The TS-5000Z taste sensor device was used to evaluate the flavor. The fact that all of the CDs, in the order of their stability constants, reduced (*p* < 0.001) the response of the taste sensor to PB in the taste assessment shows that the CDs concealed the flavor of PB by creating inclusion complexes with PB. A CD-Povidone K30 ternary system was developed. This matched the taste rating of very mildly bitter given by a human taste panel. However, a strong taste-masking effect is produced when PB is complexed with α-CD at a 1:1 molar ratio, which reduces the bitter flavor of PB by around 30%. Furthermore, it is anticipated that a larger amount of CD would have a poorer taste-masking effect in order to ensure that the majority of the PB is complexed. As a result, the α-CD-PB system is more suited for administration, especially via nasogastric or gastrostomy tubes, due to its superior water solubility and substantially lower API:CD molar ratio, which suggests a less bulky dosage. It is also known that the lower oral solubility of the bad-tasting medication molecule can be used to conceal flavor by forming complexes with CD. The β- and γ-CD complexes might be helpful in this area as well because they are insoluble and tasteless when administered orally, but they can release PB farther down in the gut. The constantly increasing number of works (Figure 5) describing the application of cyclodextrins as taste-masking agents highlights the potential of these already widely recognized cyclic oligosaccharides as valuable excipients.

## 9. Methodology

### 9.1. Study Design and Search Strategy

Two independent examiners (Ł.S. and L.A.) were chosen to select the articles. As a result, the examiners performed an extensive literature search in the databases of PubMed, Web of Science, Scopus, and the Cochrane Library. “Cyclodextrin”, “taste masking”, and “taste” were the search phrases. The search technique included a variety of terms and was purposefully wide. Articles that met the criteria were reviewed for any additional pertinent research that may have been cited and included in our analysis. Additionally, a manual search of other relevant articles on this topic was carried out. The same two independent examiners chose and categorized the papers as being included in or removed from the review based on the titles and abstracts. Duplicate articles were removed using the Rayyan for Systematic Reviews program. Following the completion of the eligibility stage, the data were taken from the selected publications. The studies were examined and discussed. Before moving on to the following phases, any disagreements during the procedure were settled by reaching an agreement.

### 9.2. Study Selection and Criteria

Two reviewers (Ł.S. and L.A.) separately went through all of the imported papers in the Rayyan program as part of the screening procedure. The use of cyclodextrins as taste-masking excipients for pharmaceutical purposes was the inclusion criterion for this review. The language and research design (including the inclusion of in vitro and in vivo experiments) were not constrained. Review papers, case studies, letters, comments, and conference abstracts were all excluded. Any discrepancies were settled by the two reviewers’ unanimous agreement once the inclusion and exclusion processes were complete.

## 10. Conclusions

Due to the ongoing development of pediatric medications and quick-release oral formulations (ODTs), drug manufacturers are paying an increasing amount of attention to the flavor of drugs. The majority of APIs have an unpleasant taste, which, if unmasked, significantly lowers patient compliance, particularly in children, making the development of taste-masking technology necessary.

Due to the variety of existing native and substituted CDs, as well as possible guest–host molar ratios in the inclusion complexes, the application of CDs is not straightforward. Also, sometimes, ternary complexation consisting of an API, cyclodextrin, and an appropriate polymer can be utilized as an alternative approach for effective taste masking. Nevertheless, as the reviewed works suggest, this solution can be an effective, safe, and inexpensive method of taste masking for pharmaceutical purposes.

## Figures and Tables

**Table 1:** Number of works describing the use of particular cyclodextrins for taste-masking purposes.

CD	Number of Works
α-CD	10
β-CD	36
γ-CD	9
HP-β-CD	28
HP-γ-CD	1
HP-α-CD	1
2,6-O-Dimethyl-β-CD	1
6-Glucosyl-β-CD	1
6-Maltosyl-β-CD	1
Sulfobutyl-ether-β-CD	1
Carboxymethyl-β-CD	1

**Table 2:** Typical taste evaluation panel used in studies of cyclodextrins as taste-masking agents. Adapted from [25], licensed under CC BY 4.0.

Parameters	Score of Taste Evaluation				
	1	2	3	4	5
Bitterness	Etremely bitter	Highly bitter	Acceptable	Very slightly bitter	Not at all bitter
Sweetness	Not at all sweet	Very slightly sweet	Acceptable	Highly sweet	Extremely sweet
Mouth feel	Very gritty	Gritty	Acceptable	Creamy	Very creamy
Flavour	Very unpleasant	Unpleasant	Acceptable	Pleasant	Very pleasant
Overall acceptability	Worst	Poor	Acceptable	Good	Very good

**Table 3:** Reviewed original works presenting results of the taste-masking properties of cyclodextrins. API—complexed API; CD—cyclodextrins used for preparation of complexes; taste evaluation method—method that has been used to determine the taste-masking properties; other methods—other physicochemical methods that have been used to study the obtained complexes; Ref.—reference to the literature position.

API	CD	Taste Evaluation Method	Other Methods	Ref.
Ranitidine hydrochloride	HP-β-CD	E-tongue: 0.2 mL of inner solution was placed within each of these sensors, compared to 0.4 mL inside the reference electrodes.	^1^H NMR	[30]
Famotidine	β-cyclodextrin	Twelve healthy human volunteers were used in a study on taste perception. They were instructed to taste 2.5 mg of the items for 5 s after they were left in their mouths. The following statements were then presented to the volunteers: 1. “A bitter taste is present”. 2. “I sense something, but I can’t place the flavor”. 3. “I don’t taste anything”.	PXRD, DSC, FTIR, UV-VIS, PS, SEM,	[31]
Famotidine	β-cyclodextrin	Twelve male or female healthy human volunteers between the ages of 23 and 27 held about 3 mL of each sample in their mouths for 10 s. The degree of bitterness was measured after expectoration. Using a numerical scale, the following values were used: 0, tasteless; 1, mildly bitter; 2, moderately bitter; 4, moderately to strongly bitter; 5, very bitter; and 6, very strongly bitter. Testing of random samples was used to validate this numerical scale. To remove bias, the mouth cavity was washed three times with distilled water. The time between testing various samples was 10 min.	PS, ^1^H NMR, DSC, PXRD, UV-VIS	[32]
Famotidine	sulfobutyl-ether-β-cyclodextrin, carboxymethyl-β- cyclodextrin	Eight healthy human volunteers between the ages of 23 and 27 were instructed to hold the reference solutions in their mouths for 10 s and report on the concentrations and bitterness levels. The following values were utilized on a numerical scale: 0 = unappealing; 1 = mildly bitter; 2 = moderately bitter; 3 = bitter; and 4 = very bitter. Testing of random samples was used to validate this numerical scale. To remove bias, the mouth cavity was washed three times with distilled water.	PS, ^13^C NMR, HPLC	[33]
Propantheline bromide, oxyphenonium bromide	α-, β-, γ-cyclodextrin, 2,6-O-dimethyl-β-CD, HP-β-CD, 6-glucosyl-β-CD, 6-maltosyl-β-CD	The sensory test comprised five subjects. These panelists tasted 35 mL of an aqueous sodium bromide solution with 154 mM sodium content, OB or PB alone, or a combination of a masking agent and either 4 mM OB or 1.5 mM PB. The following scores were used to assess how bitter these solutions were: no bitter flavor at 0; a very slight bitter taste at 1; a minor bitter taste at 2; a noticeable bitter taste at 4; and an exceedingly bitter taste at 5. For subsequent investigation, the average bitter taste intensity among the five individuals was utilized. In most cases, the standard deviations of the bitter taste intensities were about 0.7.		[34]
Prednisolone	β-cyclodextrin	Ten healthy human participants between the ages of 20 and 25 were chosen. Prednisolone solutions in pH 6.8 phosphate buffer at 10, 20, 30, 40, and 50 lg/mL concentrations were created. The volunteers judged the taste on a scale of 0 to 4 (0, no bitterness; 1, threshold bitterness; 2, bitter; 3, moderate bitterness; and 4, intense bitterness) after holding 10 mL of each solution in their mouths for 60 s.	FTIR, UV-VIS	[35]
Dextromethorphan hydrobromide, loperamide hydrochloride, cetirizine hydrochloride	HP-β-CD	Electronic taste assessment.	PS	[36]
4-Phenylbutyrate	α-, β-, and γ-cyclodextrin	The TS-5000Z taste sensor device was used to evaluate flavor. First, it was discovered that PB concentration and sensor response are related. The sensors’ reaction to the CDs was then evaluated. The reference solution underwent a sensor measurement first, and then the sample was examined.	PS, HPLC, CD, ^1^H NMR	[37]
Lidocaine hydrochloride	HP-β-CD	Electronic tongue: Using a 48-position autosampler and 25 mL beaker samples, taste was evaluated using an Alpha MOS ASTREE e-tongue system that was equipped with Alpha M.O.S. sensor set no. 2. Periods of 120 and 180 s, respectively, were set as the acquisition and analysis times. The seven sensors (ZZ, AB, BA, BB, CA, DA, and JE) were used to measure the e-tongue signal of each solution at equilibrium.	PS, DSC, FTIR, PXRD, ^1^H NMR, SEM, HPLC	[38]
Indomethacin, furosemide	HP-β-CD	E-tongue: Each sample required 100 mL of liquid, and each sample was run five times, with the first two runs serving to prepare the membranes and the remaining results being discarded. The findings were presented as a change in the membrane potential measured in millivolts (mV) with respect to the corresponding reference solution corresponding to the particular sensor.	UV-VIS, PS	[39]
Spironolactone	HP-β-CD	A total of 24 healthy participants between the ages of 18 and 40 were randomly assigned 10 mL samples that were labeled with a special three-digit number. There were 11 females and 13 men in the group. Each sample was evaluated twice. The participants had to spit the sample into a container that was given after rinsing their mouths with the samples for 5 s to cover all oral surfaces. They were to score the taste using a computerized survey that contained a 100 mm horizontal visual analog scale (anchored from “not aver-sive” to “extremely aversive”) as soon as the sample was spat out. They were asked to offer free-form descriptions of taste and scent. Participants cleaned their mouths with mineral water before and after each sample, and they were allowed to eat unsalted crackers to acclimate their palates. An interval of up to 10 min was allowed between each sample such that the one before it was no longer noticeable. If necessary, participants might instantly taste each item again.	UV-VIS, ^1^H NMR, HPLC	[40]
Diltiazem hydrochloride	β-cyclodextrin	Five members of a panel completed a sensory evaluation of the drug-CD complex sample with regard to the bitter taste, categorizing the bitter taste into the following five groups. Class 5: extremely bitter flavor; Class 4: extremely bitter flavor; Class 3: slightly bitter flavor; Class 2: a little bitter flavor; Class 1: no sour flavor. The usual control medication, which had a mean bitter taste of 5.0, was employed.	PS, UV-VIS, IR, PXRD, DSC	[41]
Captopril	β-cyclodextrin	Six healthy volunteers were instructed to ingest one CAP pill for 20 s while recording their feelings. They were instructed to spit out the contents and rinse their mouths with water after 20 s. The following taste qualities were to be ranked from 1 to 5: bitter flavor, grittiness, other taste, lingering taste in mouth, acceptance, and sulfurous taste. After 30 min, they repeated the process with CAP-CD tablets. The primary aim was the determination of a bitter taste, with other metrics serving as supplementary endpoints. Taste was rated using a numerical scale with the following values: 0 for tasteless, 1 for pleasing, 2 for mildly sweet, 3 for slightly bitter, 4 for moderately bitter, and 5 for extremely bitter.	PXRD, FTIR, DSC, SEM	[42]
Promethazine hydrochloride	HP-β-CD	A taste panel (n = 6) evaluated the acceptability of the flavor by holding a film sample containing 25 mg of medication in the mouth until it disintegrated and then spitting it out and recording the amount of bitterness: the scale ranged from 0 (no bitterness) through 0.5 (threshold bitterness), 1, 2, and 3 (severe bitterness).	FTIR, PXRD, SEM	[43]
Promethazine hydrochloride	β-cyclodextrin	E-tongue: The samples were put in a beaker with the instrument electrodes attached, and 10 cc of distilled water was used to dissolve them. Before recording the outcome, the instrument was allowed to operate for 10 min. The outcomes were determined using a scale of 1 to 10. The scale was used to evaluate the e-tongue results. Taste scale: 1 very sweet; 2–3 sweet; 4–5 sweet; 10 acceptable, really bitter.	PXRD, ^1^H NMR, ESI-MS, FTIR, DSC, SEM, PS	[44]
Triclosan	HP-β-CD	Films of formulas F8 and F10 were tested for tongue feel, bitter taste masking, mouth freshening, and in vivo dissolution time in oral cavities of healthy human volunteers (n = 9; 7 males and 2 females) with their consent. The participants were instructed to put the film on the tongue. Later, the mobility of the tongue was not restricted for the volunteers.	DSC, FTIR, PXRD, ^1^H NMR	[45]
Vardenafil	β-cyclodextrin	On a scale of 0 to 3, six healthy human volunteers were asked to judge how bitter the improved recipe tasted. When the score was less than 1, the taste was tolerable, but when it was greater than 1, the tablet’s flavor was bitter and intolerable.	FTIR, HPLC	[46]
Cefixime trihydrate	β-cyclodextrin	Twelve healthy human volunteers cleaned their mouths with 200 mL of water before placing the film dosage form on their palates (the upper part of their mouths) with their tongues. They were to carefully avoid biting the film. They were then given a CFX ODF that had been taste-masked. The time–intensity approach was used to examine the film’s flavor. After the experiment, the volunteers washed their mouths with water without ingesting any of the film’s dissolved or disintegrating components.	PS, FTIR, UV-VIS, SEM	[47]
Cefixime trihydrate	HP-β-CD	In a study to gauge the smell of CEF formulations, ten (n= 10) healthy individuals with ages ranging from 18 to 28 (mean age = 22) were enrolled as adult sensory panelists. The “facial expressions scale” or Facial Affective Scale, FAS [47], which is typically used for taste evaluation but has been modified to assess olfactory perception, was used to rate each participant’s level of contentment.	PS, UV-VIS, ^1^H-NMR	[48]
Lomefloxacin hydrochloride	HP-β-CD	No detailed information provided.	XRD, UV-VIS, FTIR	[49]
Cefuroxime axetil	β-cyclodextrin	In vitro taste assessment study: A volumetric flask containing 10 mL of phosphate buffer was added with an optimal inclusion complex amount equal to 100 mg of cefuroxime axetil. The mixture was vortexed for 30 s, filtered, and subjected to a UV–Visible spectrophotometer analysis to determine the concentration of cefuroxime axetil at 281 nm, which was then compared to the threshold value. The amount of medication that is solubilized within 30 s should not be more than the medication’s threshold bitterness concentration in order to provide appropriate taste masking.	PS, FTIR, DSC, PXRD, UV-VIS	[50]
Cefuroxime axetil	HP-β-CD	Ten human volunteers were used to assess the oral suspensions’ flavors. The study employed the pure medicine cefuroxime axetil as the benchmark, and it also compared the formulations using a market sample. Four criteria were used to classify formulations: (1) no bitter taste or flavor that has been covered up; (2) slightly bitter; (3) bitter; and (4) very bitter.		[51]
Cefuroxime axetil	HP-β-CD	Ten human volunteers were used to assess the oral suspensions’ flavors. The pure medicine, cefuroxime axetil, served as the study’s benchmark, and the commercial product was also utilized to compare with the preparations. Four criteria were used to classify formulations: (1) No bitterness or concealed bitterness. (2) Acceptable but a little bitter. (3) Acrid. (4) Extremely sour. To prevent carryover, volunteers were given enough water to drink and a one-hour rest in between each sample.		[52]
Oseltamivir phosphate	β-cyclodextrin	Three groups of two participants each were formed from six healthy human volunteers. Pure medication (tasting score of 4) and placebo (taste score of 1) were used as positive and negative controls, respectively, in a random order for each group to prevent bias. By placing the provided sample on their tongues, tasting it for one minute, and then properly washing their mouths with water after each sample evaluation, subjects were able to rate the degree of bitterness. Each participant evaluated the flavor of the sample using a 5-point scale that varied from 0 to 4 (0 being highly bitter, 1 being tasteless, 2 being somewhat bitter, 3 being moderately bitter, and 4 being pleasant).	PS, DSC, FTIR, XRD, SEM	[53]
Meloxicam	HP-β-CD with molar substitution 0.6	With six healthy human volunteers, in vivo disintegration time and taste evaluation was carried out. Every volunteer chose a pill at random and held it on their tongues. A numerical value was assigned to the taste, ranging from 0 (tasteless) to 3 (extremely bitter), with 1 denoting a mildly bitter flavor and 2 indicating a moderately bitter one.	UV-VIS	[54]
Meloxicam	HP-β-CD with molar substitution 0.6	With six healthy human volunteers, in vivo disintegration time and taste evaluations were carried out. Each volunteer picked a pill at random and placed it on their tongue. The flavor was assessed and given a numerical rating, ranging from 0 (tasteless) to 1, 2, and 3, depending on how bitter it was.	SEM, XRD, DSC, PS	[55]
Meloxicam	β-cyclodextrin, HP-β-CD with molar substitution 0.6	Six volunteers—two men (33%) and four women (67%) with ages ranging from 20 to 30—made up the research group. Each volunteer placed a nanofiber mat that had been loaded with MX on their tongue at random. The numerical values attributed to the flavor were 0 = tasteless, 1 = slight, 1.5 = slight–moderate, 2 = moderate, 2.5 = moderate–strong, 3 = powerful, and 3.5 = extremely strong bitterness.	PS, XRD, DSC, SEM	[56]
Meloxicam	HP-β-CD with molar substitution 0.6	Six healthy human volunteers took one tablet each and kept it on their tongues. The taste was evaluated and assigned a numerical value, i.e., 0 = tasteless, 1 = slightly bitter, 2 = moderately bitter, and 3 = strongly bitter.	SEM, XRD, DSC	[57]
lornoxicam	β-cyclodextrin	Before the test, ten human participants were advised to rinse their mouths with a cup of water (200 mL) and move the dosage with their tongues against the top portions of their mouths without biting. When the dosage disintegrated, they were also told to spit the contents out. Participants were asked to score the formulations’ first taste, aftertaste, mouthfeel, flavor, and general acceptability.	FTIR, HPLC, DSC, PXRD	[25]
Ibuprofen	β-cyclodextrin	No detailed information provided.	FTIR, DSC, UV-VIS	[58]
Aceclofenac	β-cyclodextrin	A dose of ASTMGA equal to 100 mg of aceclofenac was applied to the tongues of three healthy male volunteers while not drinking any water. After 1 min, the participants were asked to record the amount of bitterness, which served as the basis for taste rating. After each measurement, the mouth was washed. Between each test sample, there was a 30 min washout interval. The following values were utilized on a numerical scale: 0 = unpalatable, 1 = mildly bitter, 2 = moderately bitter, 3 = bitter, and 4 = very bitter.	DSC, PS	[59]
Aceclofenac	HP-β-CD	Among nine healthy human participants, after initially obtaining their informed agreement, the time–intensity approach was used to assess taste. One ODT (containing 100 mg of aceclofenac) was kept in the mouth until it completely disintegrated, whereas the aceclofenac-HP-CD equivalent of 100 mg of aceclofenac was retained in the mouth for 10 s before being spat out. A scale from 0 to 3 was used to measure the intensity of the bitterness, with 0 denoting no bitterness, 0.5 denoting threshold bitterness, 1 denoting minor bitterness, 2 denoting moderate bitterness, and 3 denoting extreme bitterness.	PS, HPLC, FTIR, DSC, PXRD	[60]
Rizatriptan benzoate	HP-β-CD	Six healthy human participants between the ages of 22 and 26 were chosen from both sexes. For 30 s, each participant kept 2 mg of the rizatriptan benzoate combination in their mouths. The panel evaluated the taste of pure rizatriptan benzoate as a benchmark. After expectoration, the degree of bitterness was noted. A numerical scale was employed, with 0 denoting a very bitter flavor, (1) denoting a moderately bitter flavor, (2) denoting a mildly bitter flavor, and (3) denoting a bland flavor. For the validation of the aforementioned scale, random sampling was used. To prevent bias, the mouth cavity was cleaned twice with water. There was a 30 min gap between testing the various samples.	FTIR, DSC, UV-VIS	[61]
Sumatriptan succinate	β-cyclodextrin	With their written agreement, 12 healthy human participants were chosen to evaluate the degree of flavor masking of created taste-masked mixtures/granules. Separately applied mixture/granules were put on the posterior lobe of the tongue for 4–6 s before being spat out, and the mouth was washed with water. The flavor and gritty feel of the dispersion were then noted.		[62]
Lamotrigine	β-cyclodextrin	Electronic tongue.	HPLC, PS, PXRD, FTIR, UV-VIS	[63]
Gabapentin	β-cyclodextrin	Ten healthy human volunteers between the ages of 22 and 27 were instructed to keep 10 mL of each solution in their mouths for 60 s while rating the flavor on a scale of 0 to 4 (0 denoting no bitterness, 1 denoting threshold bitterness, 2 denoting bitterness, 3 denoting moderate bitterness, and 4 denoting strong bitterness).	PS, UV-VIS, FTIR, DSC, PXRD, HPTLC	[64]
Aripiprazole	β-cyclodextrin	A volume of 25 mL of the appropriate, particle-free fluid was placed into each of the e-tongue test beakers. The tests were run six times in succession. The e-tongue BPM program detected and recorded the potentiometric difference produced between each individual sensor and the reference electrode.		[65]
Diphenhydramine epinastine, hydroxyzine, cetirizine, dl-chlorpheniramine	α-, β-, γ-cyclodextrin, HP-β-CD	Antihistaminic medication solutions (1.0 or 5.0 mM) mixed in water in the presence of CDs (10, 20, and 30 mM) were tested using seven healthy volunteers who were asked to rate the severity of the bitterness. In order to express the effects of CDs on the bitterness of medication solutions (5.0 mM diphenhydramine, hydroxyzine, cetirizine, and chlorpheniramine and 1.0 mM epinastine), the relative bitter score (bitter score in the presence of CDs/bitter score in the absence of CDs) was utilized. All samples were maintained in the mouth for 10 s, and the sample size was 10.0 mL. Volunteers tasted one sample before thoroughly gargling and moving on to the next.	UV-VIS, ^1^H NMR, CD, HPLC	[66]
Fluoxetine	β-cyclodextrin	Participants: This study included six healthy human participants. Each subject received a sample of a specified weight of a pure drug or an equivalent quantity of the FLX-CD complex, which they were instructed to taste, evaluate how bitter it was, and respond to after ten seconds. The reaction was rated on a scale from 0 to 4.	UV-VIS, DSC, FTIR	[67]
Donepezil	HP-β-CD	E-tongue: Using relative sensor outputs, it was possible to determine the potential difference (Vs Vr) between the test sample and reference solution as well as the CPA values, which were calculated as the difference (Vr Vr) between the reference solution’s potentials prior to and following sample assessment (change in membrane potential brought on by sample adsorption).	PS, FTIR, DSC, PXRD	[68]
Paroxetine hydrochloride	HP-β-CD	A five-person panel performed a gustatory sensory evaluation of a sample of a drug-HP-CD complex (1:1 molar ratio), categorizing the bitter taste into the following five categories: Class 1: no bitter taste/tasteless; Class 5: very strongly bitter; Class 4: strongly bitter; Class 3: moderately bitter; Class 2: slightly bitter.	UV-VIS, FTIR, PXRD, DSC	[69]
Atomoxetine hydrochloride	HP-β-CD	Six healthy, trained volunteers—three men and three women, ranging in age from 26 to 38—took part in this study. Both a pure atomoxetine HCl solution and an atomoxetine HCl/HP-CyD solution, both of which contained around 1.028 mM atomoxetine HCl, were made. The subjects took all of the solutions at random and kept them in their mouths for 30 s before rating the amount of bitterness on a scale of 0 to 4, with 0 denoting no taste, 1 denoting threshold, 2 denoting mildly bitter, 3 denoting bitter, and 4 denoting extremely bitter.	DSC, PXRD, SEM, ^1^H NMR	[70]
Primaquine phosphate	β-cyclodextrin	For a quinine taste sensitivity test, 20 healthy human participants between the ages of 23 and 27 were chosen. Each volunteer held 1 cc of the dispersion in their mouth for 30 s immediately after its preparation. After expectoration, the degree of bitterness was noted. The following values were placed on a scale using numbers: taste thresholds ranged from 0 (tasteless) to 3+ (extremely strong), with 0.5 (very faintly bitter) being the mildest and 3+ (severely bitter) being the strongest.	FTIR, DSC, PXRD, UV-VIS	[71]
Artemether	β-cyclodextrin	The sensory test had twenty people. For 15 s, a physical mixture or kneaded system weighing 1 g was disseminated in 50 mL of water. Each subject held approximately 1mL of the dispersion in their mouth for 30s right after its preparation. The degree of bitterness was measured after expectoration. The control was the pure drug. The following values were placed on a scale using numbers: taste thresholds range from 0 (tasteless) to 3+ (extremely strong), with 0.5 (very faintly bitter) being the mildest and 3+ (severely bitter) being the strongest. The greatest number of volunteers who described the taste as bitter or mildly bitter was used to define the threshold of bitterness.	DSC, SEM, PXRD, FTIR, PS, UV-VIS	[72]
Cetirizine hydrochloride	HP-β-CD and HP-γ-CD	For the study, 30 participants between the ages of 18 and 63 (mean age: 38.7; 66.7% female) were enrolled. The participants were asked to score the product’s flavor, sweetness, and bitterness after 10 s in the first three questions. They next cleaned their mouths and were instructed to spit out any leftovers. The second questionnaire (Q4–Q13) covered topics such as general acceptability, intensity, flavor, sweetness, and bitterness, as well as mouthfeel and texture, disintegration time, and aftertaste. Five minutes later, the panelists were tasked with answering questions 14 and 15 on the aftertaste and how long it lasted and whether they liked or disliked the product. E-tongue: 100 mL of water was used to dilute pure cetirizine HCl and a variety of cetirizine HCl formulations, which were then analyzed using the Insent1. To simulate the amount of saliva that could be present in the mouth, the number of tablets put into the water was equal to one single dosage in 5 mL of water. Using the multivariate data analysis program Simca P+/−V12, principal component analysis (PCA) was carried out to depict the findings of all seven sensors in a two-dimensional format.	HPLC	[29]
Cetirizine hydrochloride	HP-β-CD	The amount of bitterness was assessed after a film sample containing 10 mg of medication was kept in the mouth for 5–10 s before being spat out by a taste panel of human volunteers (n = 6). Following the injection of the medication and the sample, the participants were instructed to gargle with distilled water. A scale of + = highly bitter, ++ = mild to bitter, +++ = somewhat bitter, ++++ = tasteless/taste-masked, and +++++ = great flavor masking was used to indicate the degree of taste masking.	FTIR, UV-VIS, DSC, PXRD	[73]
Cetirizine hydrochloride	β-cyclodextrin	In the 24- to 34-year-old age range, 15 human participants were selected. For five seconds, volunteers were instructed to taste items. The volunteers then recorded the amount of bitterness after the samples were spat out. The following numbers were used to represent the different levels of bitterness: 0 = tasteless, 0.5 = very little bitterness, 1 = slightly bitter, 1.5 = slightly to moderately bitter, 2 = moderately bitter, 2.5 = moderate to strong bitterness, 3 = strong bitterness, and 3+ = extremely strong.	^1^H NMR, FTIR, PXRD, SEM, TD-GC-MSD	[74]
Cetirizine dihydrochloride	β-cyclodextrin	Six healthy human participants—of either sex—between the ages of 20 and 35 were chosen from a pool of twenty volunteers based on results of a taste sensitivity test. Six healthy participants were taught to identify the amount of bitterness in CTZ. To assess and identify the amount of bitterness in CTZ, caffeine was employed as the benchmark. To compare with an inclusion complex corresponding to 10 mg of CTZ and its PM, CTZ powder was put in the mouth for 15 s. The bitterness level was measured following expectoration, and the samples were then spat out. In this study, the bitterness was graded using an unstructured line scale with values ranging from 0 (no bitterness) to 12 (severe bitterness), as has been previously described.	FTIR, DSC, PXRD, ^1^H MR,	[75]
Cetirizine dihydrochloride	α-, β-, and γ-cyclodextrin	No detailed information provided.	FTIR, UV-VIS, HPLC	[76]
Cetirizine dihydrochloride	α-, β-, and γ-cyclodextrin	Thirteen healthy participants reported the bitterness tasted after having their mouths washed with 5 mL (1 mg/mL cetirizine) cetirizine-CD solutions for 10 s. There were 10 men and 3 women among the volunteers. The solutions were then expelled (spat out). The subjects carefully washed their mouths with water three minutes after tasting each sample. The levels of bitterness on the scale were as follows: 1 indicates no bitterness, 2 indicates mild bitterness, 3 indicates moderate bitterness, 4 indicates bitterness, and 5 indicates high bitterness.	UV-VIS, NMR	[77]
Levocetirizine dihydrochloride	HP-β-CD	E-tongue: Using a random sample sequence, e-tongue tests were conducted at constant ambient temperature (25 ± 0.5 °C). For the cleaning of the sensors in between measuring the samples, distilled water was utilized. Nine measurements of each sample were made under the following test conditions: 100 mL sample volume, 120 s sample collection time, and 20 s cleaning time.	SEM, UV-VIS	[78]
Levocetirizine dihydrochloride	HP-β-CD	The authors chose 10 human subjects. A complex made up of around 5 mg of drug equivalent was put on the tongue and tasted both immediately and after 20 s. By averaging the ratings of each volunteer, the overall assessment and bitterness level were reported.	UV-VIS	[24]
Levocetirizine dihydrochloride	HP-β-CD	Six healthy female volunteers between the ages of 35 and 48 were chosen to rate the drug’s level of bitterness on a scale of 0 to 5. A score of 0 indicated no bitterness, while a score of 5 suggested a highly bitter taste. The volunteers were instructed to keep a low dose of the medication (5 mg) in their mouths at the back of the tongue for 10 s while recording their results.	FTIR, DSC, UV-VIS	[79]
Levocetirizine dihydrochloride	HP-β-CD	Twelve healthy adult male human participants between the ages of 19 and 22 years old participated in taste assessment research (gustatory sensation test) to assess the mouthfeel, palatability, and in vivo disintegration time in the oral cavity for the F19 formulation. During the test, the subjects were instructed to limit their tongue movements. The reference product was first given to the participants and held in their mouths until it crumbled. After that, the participants spat out the dispersion and gargled with water. The participants assessed the test and reference formulations’ bitterness as 0—good; 1—not bitter; 2—bitter; or 3—very bitter.		[80]
Bromelain hydrolysate	β-cyclodextrin	Quantitative descriptive analysis (QDA): Using caffeine as a reference solution, samples were assessed by fifteen panelists who had completed a three-week training program. The panelists were given caffeine solutions at six different concentrations (0 to 1000 ppm, spaced 200 ppm apart). These concentrations were to be ranked by each panelist from least to most bitter. At the conclusion of the training, the concentration with the mildest bitterness was determined and utilized to assess the hydrolysate samples. Panelists were positioned in individual booths for the duration of the evaluation session. Warm water and citrus water at 0.04% (*w*/*v*) were given to the participants. The degree of bitterness was measured and recorded on a 15 cm line scale that ranged from “none” to “very bitter.”	HPLC, GC-MS, FESEM	[81]
Chitosan	α-, β-, and γ-cyclodextrins	Twelve volunteers, six males and six females, 23 to 42 years old, were asked to score the bitterness intensity on a 5-point scale ranging from “like water” (0) to “exceedingly bitter” (5) and passing through “very faintly bitter” (1), “faintly bitter” (2), “definitely bitter” (3), and “strongly bitter” (4).	^1^H NMR, FTIR, ^13^C NMR, ESI-MS	[82]
Allicin	α-cyclodextrin	The entirety of the planned research involved 18 participants, who were all between the ages of 20 and 45, in excellent health, and free of any discomfort or allergies and had the capacity to articulate and describe their sensations clearly. The volunteers were given equal portions of the physical mixture and allicin@-CD, which they were instructed to hold in their mouths for 10 s before exhaling. After this, the volunteers’ mouths were rinsed with plain water or made to feel better by chewing gum or using mouthwash (5–10 min later) while also sufficiently resting (by lying down in a ventilated area until the previous sample had no effect on tasting or sniffing) to prevent cross-contamination between samples. Volunteers were asked to rate the samples’ strength of taste and smell and respond to the following questions both during and after the evaluation: (i) Would you kindly outline each group’s preferences? (ii) Which group (within 5 s) had a greater garlic flavor at first taste? (iii) Which group exhibited a stronger garlic flavor in the latter stages (5–10 s)?	HPLC, FTIR, PXRD, ^1^H NMR, TGA	[83]
Arundic acid	α-, β-, γ-cyclodextrin, HP-α-CD and HP-β-CD	Five healthy participants participated in the gustatory sensation tests and were instructed to concentrate on the astringency and discomfort without being distracted by the fragrance. Quinine hydrochloride concentrations utilized as standards were 0.03, 0.1, 0.3, and 1 mM, and the associated bitterness and irritation ratings were 0, 1, 2, and 3, respectively.	UV-VIS, HPLC, ^1^H NMR, PXRD, PS	[84]
Bitterness suppressants of berberine hydrochloride	β-cyclodextrin	E-tongue: The solution’s total volume was 100 mL. The sensors were cleaned for 90 s in a purification bath before being submerged in a reference solution for a further 120 s of cleaning. The second reference solution was used after that for 120 s. After a 30 s equilibration period, the sensors were reset to zero. The testing started 30 s after establishing equilibrium. The sensors were briefly cleaned in each of the two reference solutions for three seconds before being put in a new reference solution to track aftertaste for thirty seconds. There were four iterations of this cycle.		[85]
Bitter gourd extract (BGE)	β-cyclodextrin	The bitterness of samples was assessed by a trained panel (n = 10: 6 males and 4 females). Student panelists between the ages of 18 and 38 were present. Each panelist received 8 samples in total, including the extract without the bitterness-masking agent (the control sample) and the extract with the bitterness-masking agent (7 samples), as part of the evaluation process for bitterness. The participants were instructed to taste each sample and assess the level of bitterness on a 10 cm unstructured scale with “weak” and “strong” labels at the left and right extremes.	FTIR, ^1^HNMR, TGA	[86]
Soybean meal	α-, β-, and γ-cyclodextrin	Electronic tongue: Before being given to tasters, samples were assigned randomly generated numbers that they were instructed to sort in order of increasing bitterness. By designating note 1 for the least bitter sample and note 3 for the most bitter sample, the findings were examined.	SEM, TGA, DSC, ^1^H NMR, PXRD	[87]
Beany off-flavors in plant-based meat analogs	α-, β-, γ-cyclodextrin	Five Amano En-zyme Inc. staff members who had received sensory assessment training evaluated the flavor and aroma of plant-based patties. In taste testing, the items were judged on the basis of five characteristics: umami, bitterness, saltiness, and sweetness. The samples were assessed for two characteristics—odor and fragrance—in sniff tests. The samples were scored on a scale ranging from 1 (weak) to 5 (strong).	HPLC, HS-SPME-GC/MS, TPA	[88]
Goat’s milk	β-cyclodextrin	Trained panelists of both sexes (n = 21) were recruited. Three samples of goat’s milk were heated up to 50+/±18 C. One sample was designated as the standard (no β-cyclodextrin), and the other two had β-cyclodextrin incorporated. In order to determine whether the number of correct responses for the number tested was equal to or greater than the number indicated in the Table of the Critical Number (Minimum) of Correct Answers at 5, 1, and 0.1% levels of significance, the results were expressed as the number of correct responses (correctly identified) and the number of total responses.	DSC, ^1^H NMR	[89]

HP-β-CD—2-hydroxypropyl-β-cyclodextrin; PS—phase solubility studies; UV-Vis—ultraviolet–visible spectroscopy; NMR—nuclear magnetic resonance spectroscopy; HPLC—high-performance liquid chromatography; CD—Circular Dichroism; SEM—scanning electron microscopy; DSC—Differential Scanning Calorimetry; PXRD—powder X-ray diffraction; FTIR—Fourier transform infrared spectroscopy; TGA—thermogravimetric analysis; GC-MS—gas chromatography–mass spectrometry; FESEM—field-emission scanning electron microscope; TD-GC-MSD—thermal desorption gas chromatograph mass spectrometer; FDSF—fast-dissolving sublingual film; ESEM—environmental scanning electron microscopy; HPTLC—high-performance thin-layer chromatography; ESI-MS—electron spray ionization–mass spectroscopy; SR-FTIR—Synchrotron Radiation Fourier Transform Infrared; HS-SPME-GC/MS—headspace solid-phase microextraction–gas chromatography/mass spectrometry.

**Table 4:** Results of application of β-cyclodextrin to mask the taste of rizatriptan benzoate. The molar ratio between the CD and API has a significant impact on the results of taste masking. Used with permission of Springer Nature, from [61]; permission conveyed through Copyright Clearance Center, Inc.

Formulation	Number of Volunteers Rating the Formulation as *			
	0	1	2	3
Rizatriptan benzoate	6	-	-	-
Inclusion complex (1:1)	6	-	-	-
Inclusion complex (1:2)	-	4	2	-
Inclusion complex (1:3)	-	-	-	6

* 0 = strongly bitter; 1 = moderately bitter; 2 = slightly bitter; 3 = tasteless.

## References

[1] Păduraru D.N., Niculescu A.-G., Bolocan A., Andronic O., Grumezescu A.M., Bîrlă R. (2022). An Updated Overview of Cyclodextrin-Based Drug Delivery Systems for Cancer Therapy. Pharmaceutics.

[2] Rassu G., Sorrenti M., Catenacci L., Pavan B., Ferraro L., Gavini E., Bonferoni M.C., Giunchedi P., Dalpiaz A. (2021). Versatile Nasal Application of Cyclodextrins: Excipients and/or Actives?. Pharmaceutics.

[3] Aiassa V., Garnero C., Longhi M.R., Zoppi A. (2021). Cyclodextrin Multicomponent Complexes: Pharmaceutical Applications. Pharmaceutics.

[4] Kovacs T., Nagy P., Panyi G., Szente L., Varga Z., Zakany F. (2022). Cyclodextrins: Only Pharmaceutical Excipients or Full-Fledged Drug Candidates?. Pharmaceutics.

[5] Hu S., Liu X., Zhang S., Quan D. (2023). An Overview of Taste-Masking Technologies: Approaches, Application, and Assessment Methods. AAPS PharmSciTech..

[6] Taruno A., Nomura K., Kusakizako T., Ma Z., Nureki O., Foskett J.K. (2021). Taste transduction and channel synapses in taste buds. Pflugers Arch..

[7] Roper S., Chaudhari N. (2017). Taste buds: Cells, signals and synapses. Nat. Rev. Neurosci..

[8] Ahmad R., Dalziel J.E. (2020). G Protein-Coupled Receptors in Taste Physiology and Pharmacology. Front. Pharmacol..

[9] Fábián T.K., Beck A., Fejérdy P., Hermann P., Fábián G. (2015). Molecular Mechanisms of Taste Recognition: Considerations about the Role of Saliva. Int. J. Mol. Sci..

[10] Coupland J.N., Hayes J.E. (2014). Physical approaches to masking bitter taste: Lessons from food and pharmaceuticals. Pharm. Res..

[11] Banerjee S., Joshi U., Singh A., Saharan V.A. (2020). Lipids for Taste masking and Taste assessment in pharmaceutical formulations. Chem. Phys. Lipids.

[12] Baguley D., Lim E., Bevan A., Pallet A., Faust S.N. (2011). Prescribing for children—Taste and palatability affect adherence to antibiotics: A review. Arch. Dis. Child..

[13] Temussi P.A. (2009). Sweet, bitter and umami receptors: A complex relationship. Trends Biochem. Sci..

[14] Pockle R.D., Masareddy R.S., Patil A.S., Patil P.D. (2023). A comprehensive review on pharmaceutical excipients. Ther. Deliv..

[15] Santoveña-Estévez A., Suárez-González J., Vera M., González-Martín C., Soriano M., Fariña J.B. (2018). Effectiveness of Antimicrobial Preservation of Extemporaneous Diluted Simple Syrup Vehicles for Pediatrics. J. Pediatr. Pharmacol. Ther..

[16] Mostafavi S.A., Varshosaz J., Arabian S. (2014). Formulation development and evaluation of metformin chewing gum with bitter taste masking. Adv. Biomed. Res..

[17] Crini G. (2014). Review: A history of cyclodextrins. Chem. Rev..

[18] French D., Wolfrom M.L., Tipson R.S. (1957). The schardinger dextrins. Adv. Carbohydr. Chem..

[19] Szejtli J. (1998). Introduction and General Overview of Cyclodextrin Chemistry. Chem. Rev..

[20] Lewandowska I., Zielińska-Pisklak M., Szeleszczuk Ł., Pisklak D.M., Sobczak M. (2020). CYKLODEKSTRYNY—ZASTOSOWANIE W PRZEMYŚLE FARMACEUTYCZNYM. Prospect. Pharm. Sci..

[21] Aree T. (2021). Advancing insights on β-cyclodextrin inclusion complexes with SSRIs through lens of X-ray diffraction and DFT calculation. Int. J. Pharm..

[22] Mazurek A.H., Szeleszczuk Ł. (2023). A Review of Applications of Solid-State Nuclear Magnetic Resonance (ssNMR) for the Analysis of Cyclodextrin-Including Systems. Int. J. Mol. Sci..

[23] Napiórkowska E. (2023). Overview of cyclodextrins and medicinal products containing cyclodextrins currently registered in Poland. Prospect. Pharm. Sci..

[24] Devi K.N., Rao N., Priyanka K., Deepika K., Mounika G. (2015). Elimination of bitter, disgusting taste of Levocetrizine di hydrochloride by HP β -Cyclodextrin. Int. J. PharmTech Res..

[25] Krishnan S., Chockalingam V. (2015). Oral Disintegrating Tablets of Analgesic Drugs alone and in Combination for Pain Management. Asian J. Pharm..

[26] Cho S., Moazzem M.S. (2022). Recent Applications of Potentiometric Electronic Tongue and Electronic Nose in Sensory Evaluation. Prev. Nutr. Food Sci..

[27] Wasilewski T., Migoń D., Gębicki J., Kamysz W. (2019). Critical review of electronic nose and tongue instruments prospects in pharmaceutical analysis. Anal. Chim. Acta.

[28] Liu J., Zuo M., Low S.S., Xu N., Chen Z., Lv C., Cui Y., Shi Y., Men H. (2020). Fuzzy Evaluation Output of Taste Information for Liquor Using Electronic Tongue Based on Cloud Model. Sensors.

[29] Preis M., Grother L., Axe P., Breitkreutz J. (2015). In-vitro and in-vivo evaluation of taste-masked cetirizine hydrochloride formulated in oral lyophilisates. Int. J. Pharm..

[30] Chay S.K., Keating A., James C., Aliev A.E., Haider S., Craig D.Q. (2018). Evaluation of the taste-masking effects of (2-hydroxypropyl)-β-cyclodextrin on ranitidine hydrochloride; a combined biosensor, spectroscopic and molecular modelling assessment. RSC Adv..

[31] Patel A.R., Vavia P.R. (2008). Preparation and Evaluation of Taste Masked Famotidine Formulation Using Drug/β-cyclodextrin/Polymer Ternary Complexation Approach. AAPS PharmSciTech.

[32] Mady F.M., Abou-Taleb A.E., Khaled K.A., Yamasaki K., Iohara D., Ishiguro T., Hirayama F., Uekama K., Otagiri M. (2010). Enhancement of the aqueous solubility and masking the bitter taste of famotidine using drug/SBE-beta-CyD/povidone K30 complexation approach. J. Pharm. Sci..

[33] Mady F.M., Aboutaleb A., Khaled K.A., Yamasaki K., Iohara D., Taguchi K., Anraku M., Hirayama F., Uekama K., Otagiri M. (2010). Evaluation of carboxymethyl-β-cyclodextrin with acid function: Improvement of chemical stability, oral bioavailability and bitter taste of famotidine. Int. J. Pharm..

[34] Funasaki N., Uratsuji I., Okuno T., Hirota S., Neya S. (2006). Masking Mechanisms of Bitter Taste of Drugs Studied with Ion Selective Electrodes. Chem. Pharm. Bull..

[35] Basu B., Aviya K.R., Bhattacharya A. (2014). Development and characterization of mouth dissolving tablets of prednisolone. J. Pharm. Investig..

[36] Preis M., Eckert C., Häusler O., Breitkreutz J. (2014). A comparative study on solubilizing and taste-masking capacities of hydroxypropyl-β-cyclodextrin and maltodextrins with high amylose content. Sens. Actuators B—Chem..

[37] Commey K., Nakatake A., Enaka A., Nishi K., Tsukigawa K., Yamaguchi K., Ikeda H., Iohara D., Hirayama F., Otagiri M. (2022). Study of the inclusion complexes formed between 4-phenylbutyrate and α-, β- and γ-cyclodextrin in solution and evaluation on their taste-masking properties. J. Pharm. Pharmacol..

[38] Wei Y., Nedley M., Bhaduri S.B., Bredzinski X., Boddu S.H.S. (2014). Masking the bitter taste of injectable lidocaine HCL formulation for dental procedures. AAPS PharmSciTech.

[39] Alopaeus J.F., Göbel A., Breitkreutz J., Sande S.A., Tho I. (2021). Investigation of hydroxypropyl-β-cyclodextrin inclusion complexation of two poorly soluble model drugs and their taste-sensation—Effect of electrolytes, freeze-drying and incorporation into oral film formulations. J. Drug Deliv. Sci. Technol..

[40] Lopalco A., Manni A., Keeley A., Haider S., Li W., Lopedota A., Altomare C.D., Denora N., Tuleu C. (2022). In Vivo Investigation of (2-Hydroxypropyl)- β-cyclodextrin-Based Formulation of Spironolactone in Aqueous Solution for Paediatric Use. Pharmaceutics.

[41] Jagdale S., Gawali V.U., Kuchekar B.S., Chabukswar A.R. (2011). Formulation and in vitro evaluation of taste-masked oro-dispersible dosage form of diltiazem hydrochloride. Braz. J. Pharm. Sci..

[42] Musuc A.M., Anuta V., Atkinson I., Popa V.T., Sarbu I., Mircioiu C., Abdalrb G.A., Mitu M.A., Ozon E.A. (2020). Development and characterization of orally disintegrating tablets containing a Captopril-Cyclodextrin complex. Pharmaceutics.

[43] Shah J.N., Shah K.N., Mehta T.A. (2015). Hydroxy propyl β-cyclodextrin complexation of promethazine hydrochloride for the formulation of fast dissolving sublingual film: In vitro and in vivo evaluation. J. Pharm. Investig..

[44] Ganguly I., Abraham S., Srinivasan B., Madhavan V. (2014). Development of fast dissolving sublingual wafers of promethazine hydrochloride. Iran. J. Pharm. Sci..

[45] Dinge A., Nagarsenker M.S. (2008). Formulation and evaluation of fast dissolving films for delivery of triclosan to the oral cavity. AAPS PharmSciTech.

[46] Al-Gethmy H.A., Fahmy U.A., Alhakamy N.A., Ahmed O.A.A., El-Say K.M. (2019). Optimization of the Factors Affecting the Absorption of Vardenafil from Oral Disintegrating Tablets: A Clinical Pharmacokinetic Investigation. Pharmaceutics.

[47] Khan Q.-u.-a., Siddique M.I., Rasool F., Naeem M., Usman M., Zaman M. (2020). Development and characterization of orodispersible film containing cefixime trihydrate. Drug Dev. Ind. Pharm..

[48] Cirri M., Mennini N., Nerli G., Rubia J., Casalone E., Melani F., Maestrelli F., Mura P. (2021). Combined Use of Cyclodextrins and Amino Acids for the Development of Cefixime Oral Solutions for Pediatric Use. Pharmaceutics.

[49] Nanjwade V., Manvi F.V., Nanjwade B. (2013). Formulation and Evaluation of Dispersible Tablets of Lomefloxacin HCl. Int. J. Drug Dev. Res..

[50] Kaushik S., Verma R., Purohit D., Pandey P., Kumar M., Kumari B., Kaushik D. (2020). Development of binary and ternary complex of cefuroxime axetil with cyclodextrin for improving pharmaceutical characteristics. Int. J. Appl. Pharm..

[51] Prabhakaran R., Harindran J. (2016). Formulation and evaluation of taste masked oral suspension of cefuroxime axetil using hydroxypropyl-beta-cyclodextrin. Asian J. Pharm. Clin. Res..

[52] Prabhakaran R., Kunchithapatham J., Harindran J. (2015). Improvement of bioavailability of taste masked cefuroxime axetil oral suspension using hydroxy propyl-betacyclodextrin. Pharm. Lett..

[53] Sevukarajan M., Bachala T., Rahul N. (2010). Novel Inclusion Complexs of Oseltamivir Phosphate-With ß Cyclodextrin: Physico-Chemical Characterization. J. Pharm. Sci. Res..

[54] Samprasilt W., Rojanarata T., Akkaramongkolporn P., Ngawhirunpat T., Opanasopit P. (2013). Fabrication of Meloxicam taste-Masked oral fast disintegrating tablet by ion exchange resin and cyclodextrin. Thai J. Pharm. Sci..

[55] Samprasit W., Akkaramongkolporn P., Ngawhirunpat T., Rojanarata T., Opanasopit P. (2015). Formulation and evaluation of meloxicam oral disintegrating tablet with dissolution enhanced by combination of cyclodextrin and ion exchange resins. Drug Dev. Ind. Pharm..

[56] Samprasit W., Akkaramongkolporn P., Ngawhirunpat T., Rojanarata T., Kaomongkolgit R., Opanasopit P. (2015). Fast releasing oral electrospun PVP/CD nanofiber mats of taste-masked meloxicam. Int. J. Pharm..

[57] Samprasit W., Akkaramongkolporn P., Ngawhirunpat T., Rojanarata T., Opanasopit P. (2013). Meloxicam Taste-Masked Oral Disintegrating Tablet with Dissolution Enhanced by Ion Exchange Resins and Cyclodextrin. AAPS PharmSciTech.

[58] Purnamasari N., Saputra P. (2020). Evaluation of orally disintegrating tablet of ibuprofen-β-cyclodextrin inclusion complex. Int. J. Appl. Pharm..

[59] Basalious E.B., Abdullah A., Ibrahim M. (2014). Utility of Mannitol and Citric Acid for Enhancing the Solubilizing and Taste Masking Properties of β-Cyclodextrin: Development of Fast-Dissolving Tablets Containing Extremely Bitter Drug. J. Pharm. Innov..

[60] Kasliwal N., Negi J.S. (2011). Development, characterization and performance evaluation of oro-dispersible tablet containing aceclofenac hydroxypropyl-β-cyclodextrin binary system. J. Incl. Phenom. Macrocycl. Chem..

[61] Dungarwal U.N., Patil S.B. (2016). Development of orodispersible tablets of taste masked rizatriptan benzoate using hydroxypropyl β cyclodextrin. J. Pharm. Investig..

[62] Prajapati S.T., Patel P.B., Patel C.N. (2012). Formulation and evaluation of sublingual tablets containing Sumatriptan succinate. Int. J. Pharm. Investig..

[63] Kande K.V., Kotak D.J., Degani M.S., Kirsanov D., Legin A., Devarajan P.V. (2017). Microwave-Assisted Development of Orally Disintegrating Tablets by Direct Compression. AAPS PharmSciTech.

[64] Rao M.R., Bhingole R.C. (2015). Nanosponge-based pediatric-controlled release dry suspension of Gabapentin for reconstitution. Drug Dev. Ind. Pharm..

[65] Gangotia P., Nehate S., Jain H., Meshram D.B. (2019). Formulation and Evaluation of Fast Dissolving Tablets of Aripiprazole. Res. J. Pharm. Tech..

[66] Ono N., Miyamoto Y., Ishiguro T., Motoyama K., Hirayama F., Iohara D., Seo H., Tsuruta S., Arima H., Uekama K. (2011). Reduction of Bitterness of Antihistaminic Drugs by Complexation with β-Cyclodextrins. J. Pharm. Sci..

[67] Marzouk M.A., Osman D.A., Mohamed O.S. (2021). In vitro and in vivo evaluation of taste-masked orodispersible tablets of fluoxetine hydrochloride for the treatment of depression. Drug Dev. Ind. Pharm..

[68] Liu T., Wan X., Luo Z., Liu C., Quan P., Cun D., Fang L. (2019). A donepezil/cyclodextrin complexation orodispersible film: Effect of cyclodextrin on taste-masking based on dynamic process and in vivo drug absorption. Asian J. Pharm. Sci..

[69] Shinde S.V., Phatak S., Awale G., Nikam S. (2020). Development of taste masked orodispersible film containing paroxetine hydrochloride. Indian J. Pharm. Educ. Res..

[70] Li S., Zhang Y., Khan A.R., He S., Wang Y., Xu J., Zhai G. (2020). Quantitative prediction of the bitterness of atomoxetine hydrochloride and taste-masked using hydroxypropyl-β-cyclodextrin: A biosensor evaluation and interaction study. Asian J. Pharm. Sci..

[71] Shah P., Mashru R. (2008). Formulation and evaluation of taste masked oral reconstitutable suspension of primaquine phosphate. AAPS PharmSciTech.

[72] Shah P., Mashru R. (2010). Palatable reconstitutable dry suspension of artemether for flexible pediatric dosing using cyclodextrin inclusion complexation. Pharm. Dev. Technol..

[73] Mishra R., Amin A. (2013). Optimization and characterization of rapidly dissolving films of Cetirizine hydrochloride using cyclodextrins for taste masking. Int. J. PharmTech Res..

[74] Lee C., Kim S., Youn Y., Widjojokusumo E., Lee Y., Kim J., Lee Y., Tjandrawinata R.R. (2010). Preparation of bitter taste masked cetirizine dihydrochloride/β-cyclodextrin inclusion complex by supercritical antisolvent (SAS) process. J. Supercrit. Fluids.

[75] Katewongsa P., Lertsuphotvanit N., Phaechamud T. (2017). Cetirizine Dihydrochloride, β-Cyclodextrin Inclusion Complex by Ethanol Kneading for Taste Masking. Indian J. Pharm. Sci..

[76] Stojanov M., Larsen K.L. (2011). Cetirizine release from cyclodextrin formulated compressed chewing gum. Drug Dev. Ind. Pharm..

[77] Stojanov M., Wimmer R., Larsen K.L. (2011). Study of the inclusion complexes formed between cetirizine and α-, β-, and γ-cyclodextrin and evaluation on their taste-masking properties. J. Pharm. Sci..

[78] Kazsoki A., Palcsó B., Omer S., Kovacs Z., Zelkó R. (2022). Formulation of Levocetirizine-Loaded Core–Shell type nanofibrous orally dissolving webs as a potential alternative for immediate release dosage forms. Pharmaceutics.

[79] Labib G.S. (2015). Novel levocetirizine HCl tablets with enhanced palatability: Synergistic effect of combining taste modifiers and effervescence technique. Drug Des. Dev. Ther..

[80] Mahesh A., Shastri N.R., Sadanandam M. (2010). Development of taste masked fast disintegrating films of levocetirizine dihydrochloride for oral use. Curr. Drug Deliv..

[81] Normah I., Nurul Fasihah R. (2017). Evaluation of β-cyclodextrin masking effect on the bitterness of angelwing clam (*Pholas orientalis*) hydrolysate. Int. Food Res. J..

[82] Binello A., Cravotto G., Nano G.M., Spagliardi P. (2004). Synthesis of chitosan–cyclodextrin adducts and evaluation of their bitter-masking properties. Flavour Fragr. J..

[83] Zhou Y., Feng J., Peng H., Guo T., Xiao J., Zhu W., Qian W., Zhang J., Wu L. (2021). Allicin inclusions with α-cyclodextrin effectively masking its odor: Preparation, characterization, and olfactory and gustatory evaluation. J. Food Sci..

[84] Miyamoto Y., Nakahara M., Motoyama K., Ishiguro T., Oda Y., Yamanoi T., Okamoto I., Yagi A., Nishimura H., Hirayama F. (2011). Improvement of some physicochemical properties of arundic acid, (R)-(−)-2-propyloctanonic acid, by complexation with hydrophilic cyclodextrins. Int. J. Pharm..

[85] Liu R.-X., Gao X.-J., Wang J.-M., Dai L.-P., Kang B.-Y., Zhang L., Shi J.-H., Gui X.-J., Liu P., Li X.-L. (2017). Traditional human taste panel and taste sensors methods for bitter taste masking research on combined bitterness suppressants of berberine hydrochloride. Sens. Mater..

[86] Tran C.T.H., Nargotra P., Pham H.T.C., Lieu D.M., Huynh P.K., Wang H.M.D., Dong C.-D., Kuo C.-H. (2023). The effect of carboxymethyl cellulose and β-cyclodextrin as debittering agents on bitterness and physicochemical properties of bitter gourd extract. J. Food Sci. Technol..

[87] Monge Neto A.A., Ströher R., Assenha H.B.R., Scagion V.P., Correa D.S., Zanin G.M. (2017). Interaction of peptides obtained from the enzymatic hydrolysis of soybean meal with cyclodextrins: An evaluation of bitterness reduction. J. Incl. Phenom. Macrocycl. Chem..

[88] Sakai K., Sato Y., Okada M., Yamaguchi S. (2022). Cyclodextrins produced by cyclodextrin glucanotransferase mask beany off-flavors in plant-based meat analogs. PLoS ONE.

[89] Meier M.M., Drunkler D.A., Bordignon-Luiz M.T., Fett R., Szpoganicz B. (2001). The influence of β-cyclodextrin on goaty flavour—Characterization of synthetic inclusion complexes with capric acid and caprylicacid. Br. Food J..

